# Potential Biodiversity Footprint Reductions from Intended Shifts to Plant-Based Meals

**DOI:** 10.1021/acs.est.6c02675

**Published:** 2026-09-24

**Authors:** Hanzhong Zheng, Koen J. J. Kuipers, Selwyn Hoeks, Mark A. J. Huijbregts, Nicole Neufingerl, Elizabeth H. Zandstra, Sarah Sim

**Affiliations:** † Department of Environmental Science, Radboud Institute for Biological and Environmental Sciences, 6029 Radboud University, Heyendaalseweg 135, Nijmegen 6525AJ, The Netherlands; ‡ Unilever Foods Innovation Centre Wageningen, Bronland 14, Wageningen 6708WH, The Netherlands; § Division of Human Nutrition and Health, Wageningen University & Research, Stippeneng 4, Wageningen 6708WH, The Netherlands; ∥ Safety, Environmental and Regulatory Sciences, Unilever R&D, Colworth Science Park, Sharnbrook, Bedfordshire MK44 1LQ, U.K.

**Keywords:** meat, dairy, plant-based alternatives, consumer behavior change, biodiversity footprint, self-reported

## Abstract

Adopting plant-based diets can mitigate biodiversity loss by reducing the demand for land required for feed and livestock. Yet, most assessments rely on hypothetical dietary scenarios that potentially overestimate biodiversity gains. We conducted a large-scale consumer survey across three countries (NLD, GBR, and USA) and estimated the biodiversity footprints of current and intended future diets using the loss of mean species abundance (MSA-loss) and the loss of global species richness (RSR-loss) as indicators. Over half of the respondents expressed no intention to reduce meat or dairy consumption. Among those intending to replace meat with plant-based alternatives, biodiversity footprints based on MSA-loss associated with the consumption of meat and plant-based alternatives in main meals would decrease by up to 34% on average. When considering all respondents, the average reduction in MSA-loss from meat and meat alternatives in the three countries would be 13%. We found similar results for current and intended future consumption of dairy and their plant-based alternatives. RSR-loss showed the same patterns as MSA-loss, although dairy alternatives would contribute a large share of the total biodiversity footprint based on RSR-loss in the USA. Our findings show that intended dietary shifts may not match hypothetical dietary scenarios and that achieving sustainable dietary shifts to reduce biodiversity loss remains a considerable challenge.

## Graphical abstract

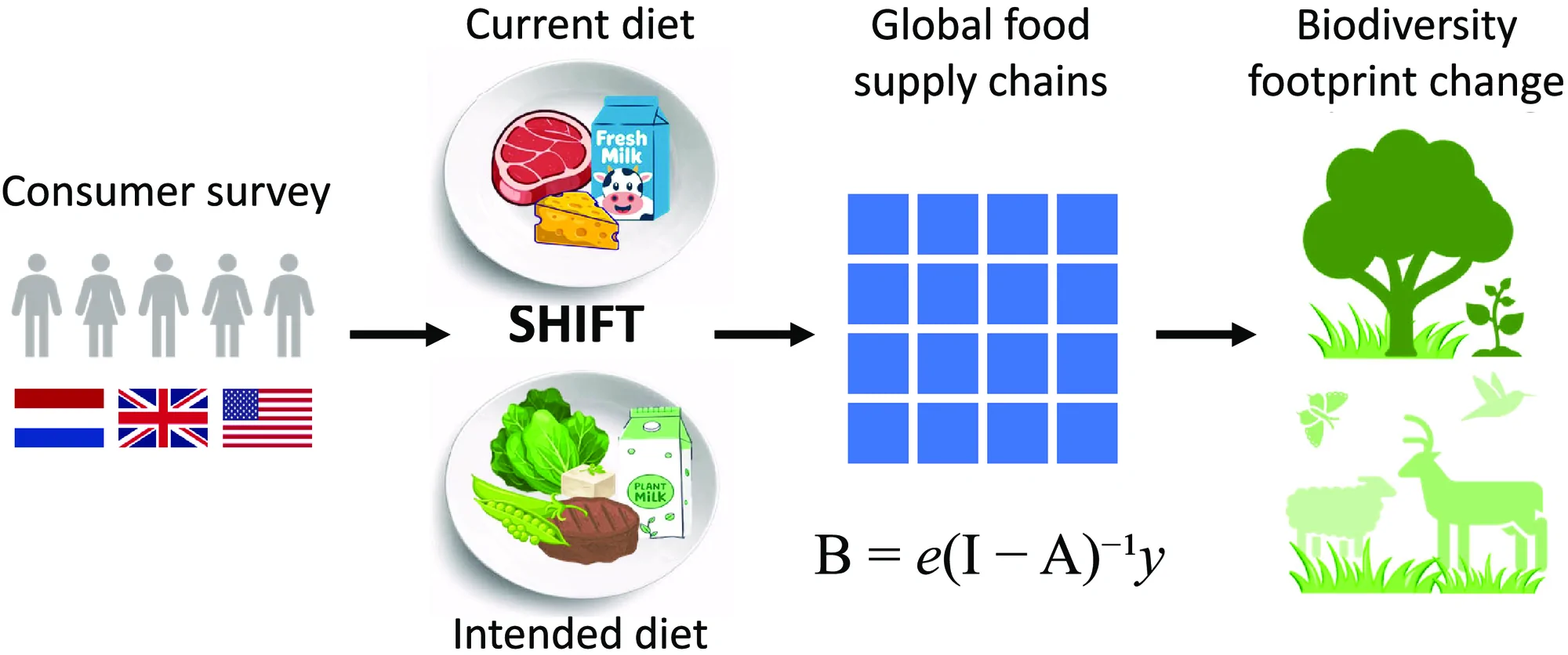

## Introduction

The global food system accounts for 33% of global anthropogenic greenhouse gas (GHG) emissions, 40% of land use, and 70% of freshwater consumption.^1–4^ Land use for crops and pasture is a major driver of biodiversity loss.^5^ The amount of cropland and pasture required for global food production is determined by population size, agricultural productivity, supply chain efficiencies and dietary patterns.^6,7^ Indeed, Kastner et al. found that dietary changes from cereals to meat and dairy in rapidly developing regions like Eastern Asia, drive land demand for food production more than population growth.^6^ These dietary changes run counter to those recommended for biodiversity loss mitigation. For instance, the EAT-Lancet Commission has proposed that a key priority for wealthier countries is to substantially reduce meat and dairy consumption.^3^

Previous studies comparing different dietary patterns (e.g., vegan, vegetarian, meat-eater) have shown the biodiversity impact of a vegan diet is on average 65% lower compared to a diet that contains high levels of animal-based products, highlighting the lower biodiversity impact of plant-based diets.^8^ Adopting plant-forward diets,^9^ which emphasize a substantial reduction in animal product consumption while increasing plant-based food consumption, has also been shown to reduce a range of other environmental impacts, such as GHG emissions and water use.^10–12^ Traditional (e.g., tofu and tempeh) and novel (e.g., meat alternatives that mimic the taste and texture of animal meat) plant-based meat alternatives are becoming increasingly popular among those seeking to adopt more plant-based ways of eating.^13–15^

Insights into the environmental benefits of dietary shifts have mostly been obtained through assessment of dietary scenarios focused on assumption-driven dietary changes or modifications needed to achieve national dietary guidelines.^16,17^ Such modeled scenarios may not accurately reflect real-world individual intentions to shift diets. For example, the extent to which meat-eaters may shift toward increased consumption of plant-based alternatives and the corresponding biodiversity impacts has not yet been investigated.^8^ Hence, there is a need for comprehensive analyses of individuals’ intention to engage in dietary behavior change to provide realistic data for environmental modeling of dietary transitions. Quantifying potential biodiversity loss mitigation from such dietary change is essential for developing targeted interventions that encourage more sustainable consumption patterns and population-wide dietary shifts.

Here, we advance the present knowledge by using individual dietary change intention survey data with biodiversity footprint assessment, providing a novel perspective on the effects of intended dietary transitions. To this end, we conducted a survey across The Netherlands (NLD), United Kingdom (GBR), and United States (USA) to quantify the current and intended near future (i.e., within the next six months) intake of meat (e.g., beef, pork, and poultry) and meat alternatives (e.g., vegetables, tofu, and plant-based meats that mimic the taste and texture of animal meat) during the main meal of the day, as well as current and intended near future intake of dairy (e.g., milk, yoghurt, and cheese) and dairy alternatives (e.g., soymilk, plant-based yoghurt, and plant-based cheese) during the day. We used the Food and Agriculture Biomass Input-Output (FABIO)^18^ model to trace both primary and processed agricultural commodity supply chains and quantify biodiversity footprints per kilogram of food commodity consumed in each country. FABIO was selected because it provides high level of agricultural commodity detail and because it considers physical flows that avoid the price distortions present in monetary input-output databases.^18^ Biodiversity footprints were assessed using two complementary indicators: mean species abundance loss (MSA-loss) to evaluate impacts on local ecosystem intactness,^19^ and relative species richness loss (RSR-loss) as a supplementary indicator for global species extinction footprints.^20^ We then combined the survey data and biodiversity footprints per food commodity to quantify the respondents’ yearly total biodiversity footprint of current and intended near future intake of meat and alternatives as well as of current and intended near future intake of dairy and dairy alternatives. This study goes beyond hypothetical dietary scenarios by quantifying biodiversity footprints of individual dietary preferences and intended changes.

## Methods

An online survey was conducted in the NLD, GBR and USA to collect data on respondents’ current and intended near future intake of meat and meat alternatives during the main meal of the day as well as current and intended near future intake of dairy and dairy alternatives during the day (survey is provided in Text Section S1). We used FABIO combined with biodiversity indicators (i.e., MSA-loss and RSR-loss, see RSR-loss results in SI) to quantify the land-use related biodiversity footprint per kg of food commodity consumed in each country. These values were applied to survey-based individual dietary intake data to estimate annual biodiversity footprints related to the current and intended near future intake of meat and meat alternatives, as well as the current and intended near future intake of dairy and dairy alternatives. We then quantified the change in biodiversity footprints as the difference between current and intended future intake of meat and meat alternatives or dairy and dairy alternatives. Finally, we explored variations in the change in biodiversity footprints across the three countries, as well as demographic differences within each country using multivariate analysis.

### Survey

The online survey was conducted in November 2023 across 2436 respondents in The Netherlands, 2079 in the GBR and 2058 in the USA. Recruitment was guided by soft quotas to obtain broadly comparable samples across countries, with approximately equal distributions across gender (male and female), three age groups (18–33, 34–49, 50–65 years), education level (low and high), and food environment for urban/rural (see Text Section S2). Respondents were asked about current and intended near future intake of meat (e.g., beef, pork, and poultry) and meat alternatives (i.e., plant-based meat alternatives that mimic the taste and texture of meat, tofu or tempeh, pulses, nuts or seeds, vegetables, cheese or dairy items, eggs, fish or seafood) during the main meal of the day. In addition, respondents were asked about current and intended near future intake of dairy (i.e., milk and dairy drinks, yoghurt/quark, cheese, ice cream, dairy desserts) and dairy alternatives (e.g., plant-based alternatives for milk and dairy drinks, yoghurt/quark, cheese, ice cream, dairy desserts) (see Table S1 for a list of products covered in survey). Respondents were recruited from an online consumer panel through a market research agency (Essensor B.V.). Panel members had previously consented to participate in survey research and were invited to complete the survey via a web-based interface. The estimated time required to complete the survey was about 15 min.

Respondents were classified into four dietary groups based on self-reported dietary restrictions toward the consumption of animal-based foods: (1) meat-eater, i.e., no restriction toward meat consumption, (2) pescatarian, i.e., excluding meat from the diet but not excluding fish, (3) vegetarian, i.e., excluding meat and fish from the diet, but not excluding dairy and eggs, and (4) vegan, i.e., excluding meat, fish, dairy and eggs from the diet. Meat-eating respondents were asked questions about their consumption, reduction and replacement of both meat and meat alternatives as well as dairy and dairy alternatives, while pescatarian and vegetarian respondents were asked questions related to dairy only. If respondents were identified as vegan, they did not proceed with dietary related questions. Their survey responses were not in scope of our analysis (detailed in the following Methods section) since the purpose of the survey was to elicit information on respondents’ intention to shift from meat intake to meat alternatives as well as dairy intake to dairy alternatives. The survey collected information on respondents’ (1) current consumption (meat and meat alternatives and dairy and dairy alternatives), (2) stages of change—whether they intend to or are already reducing their meat or dairy consumption (respondents were classified into three stages of dietary change: no intention to reduce consumption, intention to reduce consumption within the next six months, and having already initiated reductions; see Text Section S3), (3) dietary approach for reducing meat/dairy, (4) barriers to reducing meat/dairy.

### **Annual Intake** (kg per Food Product per Person per Year)

To evaluate respondents’ current meat and meat alternatives consumption, we assessed the frequency and the type of meat that was consumed during the main meal of the day over the past month. The main meal was defined as the biggest meal of the day, often the warm meal, consumed for lunch or dinner. In the survey, we provided six pictures to enable respondents to best estimate their meat portion size (i.e., the amount of meat consumed per main meal): very small (21 g), small (36 g), medium-small (75 g), medium-large (113 g), large (172 g) and very large (277 g).^21^

To investigate respondents’ approaches to (further) reduce meat intake, the survey assessed whether, and to what extent, respondents intend to reduce the consumption of meat during their main meal, and whether, and to what extent, they intend to replace it with meat alternatives in the near future. A similar framework was applied to dairy and dairy alternatives.

To quantify respondents’ current and intended future food consumption, we converted the reported frequencies for meat and meat alternatives, and for dairy and dairy alternatives into the yearly total number of portions per individual (see Text Section S4). For meat, the corresponding mass (kg per person per year) was estimated for each respondent by multiplying the yearly total number of portions with the respondents’ reported habitual portion size for meat. For meat alternatives, dairy and dairy alternatives, the corresponding mass (kg per person per year) was estimated by multiplying the yearly total number of portions with a standard portion size for each product category (see Table S1) and a correction factor. The standard portion sizes were derived based on reference portion sizes provided by national health institutes or dietetic authorities in the three countries.^22–24^ The aim of the correction factor was to account for variations in portion sizes across individuals. We assumed that respondents who reported a relatively big portion size for meat, would also replace meat with a relatively big portion of an alternative product and vice versa, and that they would also consume relatively bigger portions of dairy and dairy alternatives. Therefore, for each respondent, an individual correction factor was calculated as the ratio of the respondents’ habitual reported portion size of meat and the standard portion size for meat (i.e., 100 g, see Table S1).

Survey respondents were excluded if: (1) they selected “Other” for gender (*n* = 12), because our demographic analysis compared female and male groups; (2) they did not complete the questions required for classification into dietary groups (*n* = 430); (3) their answers produced estimates of daily food intake that were deemed contradictory (e.g., respondents classified themselves as meat-eaters, but indicated meat portions per year = 0 and meat alternatives portion per year = 0, *n* = 21). A total of 463 respondents were excluded, resulting in a final sample size of 6110.

### Biodiversity Footprints of Individual Meat, Dairy and Alternative Intake

We used FABIO 2020 to quantify the biodiversity footprints of both current and near future diets. We first estimate the global land use footprints per food commodity in the NLD, GBR and USA (i.e., considering worldwide land use corresponding to the production of food commodities consumed in NLD, GBR and USA). FABIO is a balanced and physical unit multiregional input-output database, covering 123 agriculture commodities, to which all food products listed in our survey could be mapped. Based on FAOSTAT Commodity Balance Sheets, FABIO links production, processing, and trade flows to final consumption, enabling land-use based biodiversity footprints to be traced along global food supply chains. Feed use is represented through the allocation of crop commodities to livestock sectors according to their feed requirements.^18^ FABIO land use footprints describe harvested areas. Since the physical area of agricultural land, rather than the area of harvested land drives biodiversity impacts, we converted the area of harvested land per food commodity into physical land, based on crop-and region-specific conversion ratios obtained from the Spatial Production Allocation Model for year 2010.^25^

To quantify biodiversity footprints, we linked the physical land use footprints to an impact indicator for decline in ecosystem intactness per unit of land use derived from the GLOBIO4 model, which quantifies the loss in mean species abundance (MSA),^19^ distinguishing the biodiversity impacts on plants and vertebrates. We calculated land-use related biodiversity impacts on local ecosystem intactness responses as the average of plant and vertebrate MSA responses, following Schipper et al.^19^ As a complementary indicator, we also quantified biodiversity footprints using an indicator of the relative loss in the total number of species of vertebrates and plants (RSR-loss), to capture impacts on global species richness.^20^ We only consider the biodiversity footprint of land-based food production (excluding fish farming). The biodiversity footprints of each commodity consumed in the NLD, GBR and USA was formulated as follows

1 $$B=e{\left(I-A\right)}^{-1}y$$

where ***e*** is the direct biodiversity impact intensity vector, which denotes the biodiversity loss per total production of agricultural commodity for each country. ***A*** is the global agricultural interindustry coefficient matrix, containing the amounts of outputs and inputs from one country-specific sector to another. ***I*** is the identity matrix, and (***I*** – ***A***)^−1^ is equal to the Leontief inverse matrix. ***y*** is the vector of final consumption per food item. ***B*** represents the biodiversity footprints of each food item that is consumed in the NLD, GBR and USA.

We first calculated the biodiversity footprints per kg of each food commodity consumed in the NLD, GBR and USA by dividing the biodiversity footprints of each food commodity by its total national consumption in 2020. We then applied food product conversion factors to link FABIO food commodities to survey food products (see Text Section S5) and derive the biodiversity footprint per kg of final food products. Finally, we multiplied this value with each respondent’s estimated annual consumption (in kg) of current (survey collected in 2023) and intended near future (within the next six months) meat and meat alternative intake as well as current and intended near future dairy and dairy alternative intake, we obtained the biodiversity footprints for both current and near future dietary change for each individual respondent.

### Multivariate Analysis

We applied an Extreme Gradient Boosting (XGBoost) machine learning algorithm to explore the extent to which shifts in biodiversity footprints due to dietary change differ between sociodemographic groups (based on age, gender, education, food environment (urban/rural)) and dietary behavior (portion size, annual number of portions consumed). XGBoost is a scalable and regularized tree-based ensemble learning method that is particularly well-suited for capturing nonlinear relationships and variable interactions.^26^ XGBoost is widely used in classification and regression tasks for its high predictive accuracy, efficiency, and ability to handle mixed-type variables and missing data.^26^ For tuning the hyperparameters we utilized the *caret* package (version 6.0–90) in combination with *xgboost* (version 1.7.11.1) in *R* (version 4.1.2).

First, an XGBoost classification analysis was used to identify which sociodemographic variables contribute most to predicting the probability of intention to shift diet. For each sociodemographic variable (e.g., age), we compared the respective groups (e.g., 18–33, 34–49, and 50–65 age group) to determine which are most inclined toward dietary change. Variable importance was quantified by the gain in the accuracy of the model predictions.

Second, an XGBoost regression analysis was applied to data from the group of respondents intending to reduce their meat and dairy consumption in the near future to quantify the potential average change in biodiversity footprints per sociodemographic group. Variable importance was quantified by the gain in the root-mean-square error (RMSE) when excluding the variable.

## Results

### Biodiversity Footprint of Meat, Meat Alternatives, Dairy, and Dairy Alternatives Intake

Table 1 shows the current annual consumption of meat and meat alternatives (during main meal), dairy and dairy alternatives per person per country. Across the three countries, the USA showed the highest average meat intake per person (44.49 kg y^–1^), which was substantially higher than per person meat consumption in the NLD and GBR (respectively 30.15 kg y^–1^ and 33.98 kg y^–1^). Additionally, respondents in the USA also reported the highest consumption of meat alternatives, dairy, and dairy alternatives compared to the NLD and GBR. We observed large standard deviations for dairy alternatives, which indicates substantial variation in consumption of dairy alternatives across all three countries.

**Table 1:** Mean (and s.d.) Annual per Capita Intake of Meat and Meat Alternatives (Main Meal), Dairy and Dairy Alternatives (kg per Person per Year)^a^

Country	NLD	GBR	USA
*n*	2267	1906	1937
Meat (kg per person y^–1^)	30.15 (23.19)	33.98 (23.70)	44.49 (30.11)
Meat alternatives (kg per person y^–1^)	11.13 (13.10)	14.01 (15.89)	15.57 (20.01)
Dairy (kg per person y^–1^)	153.57 (185.68)	207.96 (228.67)	220.71 (286.74)
Dairy alternatives (kg per person y^–1^)	16.60 (80.75)	29.38 (98.34)	38.53 (114.11)

a We assume that 1 L of milk equals 1 kg of milk.

Estimates of biodiversity footprints based on the average current annual intakes of meat and meat alternatives (during main meal), dairy, and dairy alternatives per country are shown in Figure 1. The per capita absolute value results for each country indicate that the USA exhibits the highest biodiversity footprint, with a mean MSA-loss•ha of 0.126 per person for meat and meat alternatives, which is 2.6 and 3.5 times higher compared to the NLD and GBR, respectively. This difference can be attributed to the higher yearly consumption of animal meat products such as beef (on average 40% more than NLD and GBR) and lamb (30% more than NLD and GBR). Meat products significantly contribute (more than 90%) to the total biodiversity footprint of meat and meat alternatives, with beef being the dominant contributor among all meat products (Figure 1b). Biodiversity footprints were distributed across both domestic production and international supply chains (Figure 1a). For the USA, a large share of impacts originated from domestic production, whereas footprints associated with consumption in the NLD and GBR were more strongly linked to imports. Results based on RSR-loss were broadly consistent with those obtained using MSA-loss, with meat products accounting for the majority of biodiversity footprints across all countries (Figure S1b). However, the sourcing countries identified using RSR-loss indicated a greater contribution from biodiversity-rich regions such as Brazil (Figure S1a,c). Biodiversity footprints of dairy consumption are highest in the USA as well (Figure 1c). This overall pattern was also observed for RSR-loss, although dairy alternatives contributed a large share of the total biodiversity footprint in the USA (Figure S1d).

**Figure 1:**
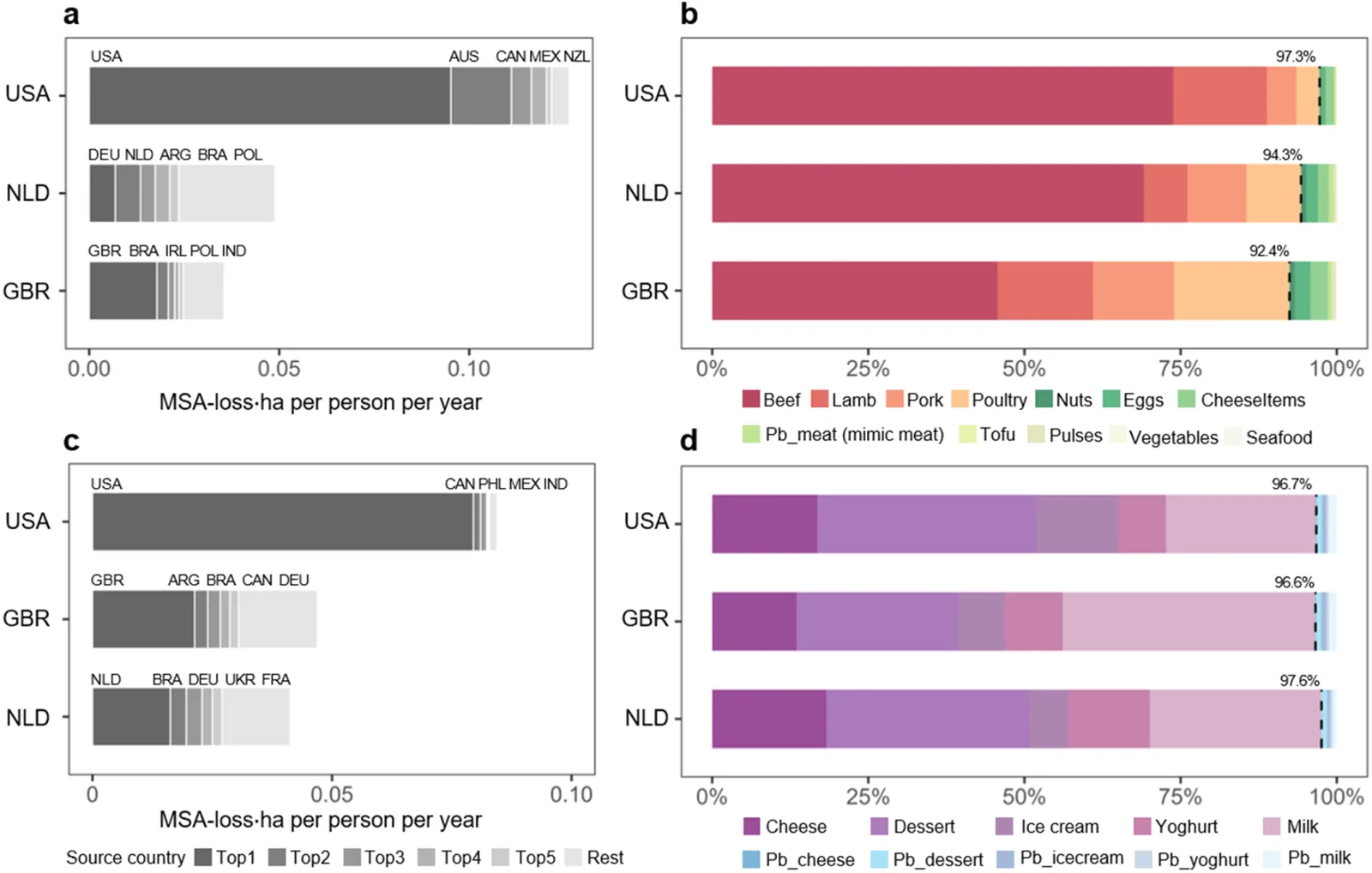
The biodiversity footprint related to average current intake of meat, meat alternatives, dairy, and dairy alternatives per person per year. (a), The absolute MSA-loss of current consumption of meat and meat alternatives by sourcing countries. (b), Contribution analysis of meat and meat alternatives to biodiversity footprint. (c), The absolute MSA-loss of current consumption of dairy and dairy alternatives by sourcing countries. (d), Contribution analysis of dairy and plant-based (Pb) dairy alternatives to biodiversity footprint. The sourcing countries in panels (a, c) show the 5 largest contributing countries, with all remaining countries aggregated as Rest. The iso3 country codes (see Table S3) displayed above each bar indicate the top 5 sourcing countries in descending order. The dashed line in panels b and d presents the separator between meat/dairy and alternatives. Biodiversity footprints are estimated based on the average of the current intake across respondents.

To better understand the variation in the current biodiversity footprint across the three countries, we observed that respondents who reported larger portion size also reported higher meat/dairy consumption and consequently higher biodiversity footprints. On average, USA respondents reported larger portion sizes of meat and alternatives (1.81 ± 0.85 times the standard portion size) compared to NLD (1.24 ± 0.71 times the standard portion size) and GBR (1.47 ± 0.72 times the standard portion size) (Figure S2), which explains the higher biodiversity footprint associated with meat and meat alternatives consumption observed in the USA in addition to the larger share of meat in their total intake of meat and meat alternatives. Respondents with the highest current consumption of meat, and therefore also the highest biodiversity footprints, are least likely to intend to reduce their meat intake in the future (Figure S2). In contrast, respondents who indicated that they had already reduced their meat consumption showed a substantially lower contribution, reflecting the potential of meat reduction behaviors to mitigate biodiversity loss. Those who indicated intention to reduce their meat intake in the near future demonstrated a variable contribution to their country’s total biodiversity footprint.

### Potential Biodiversity Loss Mitigation from Adopting Meat Alternatives and Dairy Alternatives

Figure 2 illustrates the potential biodiversity loss mitigation of shifting from meat to meat alternatives, as well as shifting from dairy to dairy alternatives, among respondents who indicated an intention to reduce meat or dairy consumption within the next six months. For respondents who intend to reduce meat consumption and replace it with meat alternatives, we calculate that there would be a consistent decrease in the biodiversity footprints of meat and meat alternatives across all countries: 0.012 MSA-loss·ha for the GBR, 0.016 MSA-loss·ha for the NLD, and 0.039 MSA-loss·ha for the USA (Figure 2). This would represent an average of 34.3% reduction when comparing biodiversity footprints of current and intended future intake of meat and meat alternatives (Figure S3). The difference in reduction potential can be explained in that, although respondents in the GBR and NLD intend to consume meat alternatives more frequently within the next six months, the average absolute meat consumption reduction is higher in the USA due to the larger portion sizes. The geographic distribution of biodiversity footprint reductions differed across countries. In the USA, most avoided impacts would be associated with domestic production, whereas reductions linked to dietary changes in NLD would be distributed across a broader set of international sourcing countries (Figure S5a).

**Figure 2:**
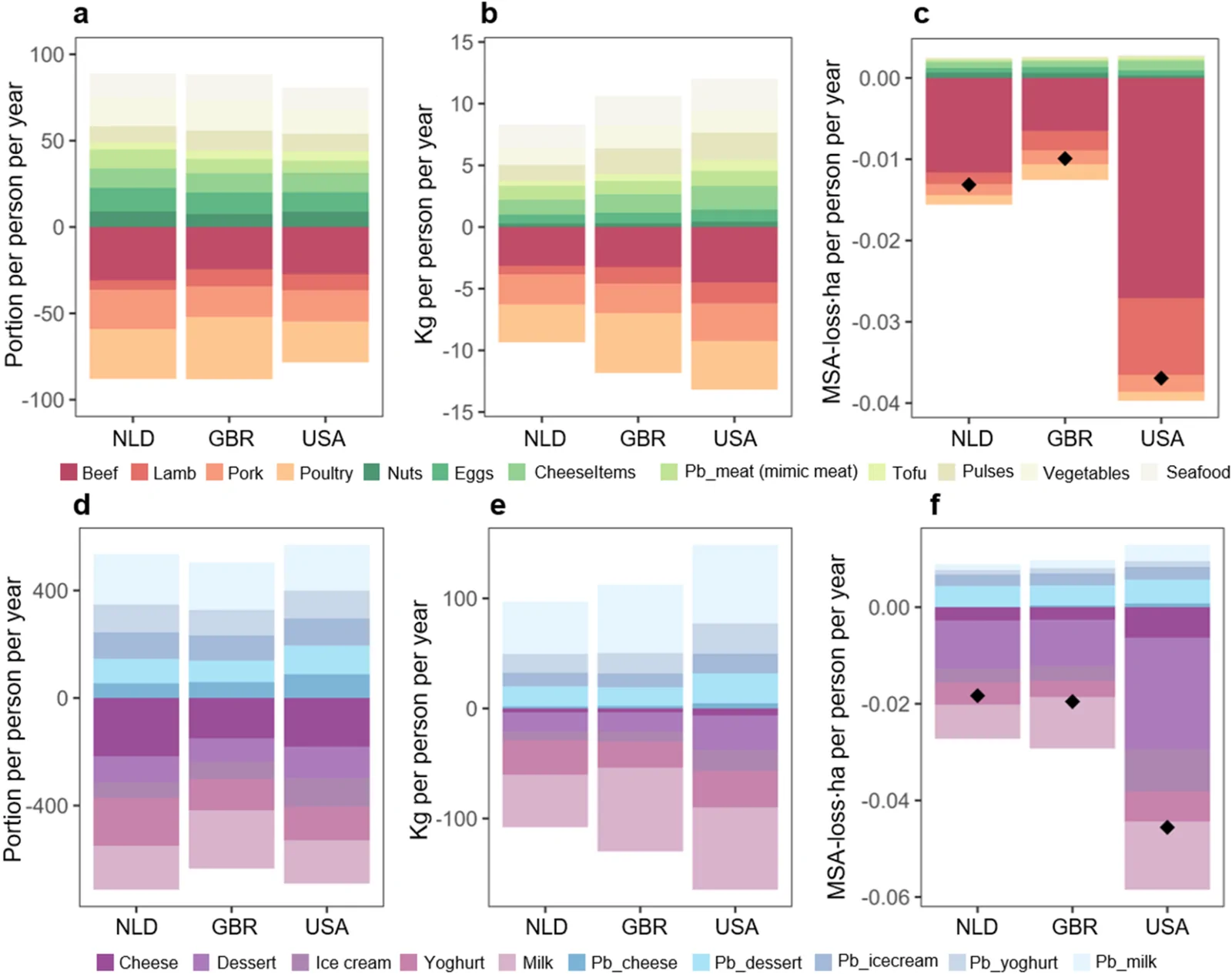
Dietary shift in the near future. (a–c) Estimated reduction of meat consumption and increased consumption of meat alternatives expressed as portions per person y^–1^ (a), kg per person y^–1^ (b), and related change in MSA-loss•ha per person y^–1^ (c) in terms to those people who indicate intention to reduce meat. (d–f), Estimated reduction of dairy consumption and increased consumption of plant-based (Pb) dairy alternatives expressed as portions (d), kg (e) and related change in MSA-loss (f) among those respondents who intend to reduce dairy intake. The portions, kg, and MSA-loss·ha are reported as the average across respondents. The diamond in the MSA-loss panel represents the net change (reduction) in MSA-loss·ha for each country.

Similarly, the potential biodiversity loss mitigation from adopting dairy alternatives demonstrate consistent reductions among respondents intending to reduce dairy consumption: 0.027 MSA-loss·ha for the NLD, 0.029 MSA-loss·ha for the GBR, and 0.058 MSA-loss·ha for the USA, equivalent to about a 50% reduction between biodiversity footprints of current and intended future intake of dairy and dairy alternatives (Figure 2 and Figure S3). Among the various dairy products, milk and desserts contribute most to reducing the biodiversity footprint when replaced by dairy alternatives. The main sourcing countries of dairy products also exhibited biodiversity footprint reductions, similar to those observed for meat products (Figure S5b). RSR-loss yielded comparable results for the NLD and GBR, however, biodiversity footprints associated with dairy alternatives would be increase in the USA, indicating that the biodiversity impacts of dietary substitutions depend on the specific alternative products consumed (Figure S4, Figure S6f, Figure S7b).

Across the three countries, respondents reported similar intentions to reduce portions of beef and poultry, with the exception of the GBR, where more respondents indicated a desire to reduce poultry. However, given that beef consumption has the highest biodiversity footprint intensity (i.e., biodiversity footprint per kilogram of beef) compared to other meat products, replacing poultry consumption offers limited potential for reducing the biodiversity footprint compared to replacing beef consumption.

### Assessing the Effect of Demographics and Current Dietary Behavior on Potential Biodiversity Footprints

We first examined the probability of intention to reduce meat or dairy intake based on demographic and current dietary behavior variables (Figure 3). Current meat consumption (meat portions per year) was the most important predictor of the probability to reduce meat intake and replace it with meat alternatives (Figure 3a). A similar pattern was observed for dairy, where current dairy consumption best predicted the probability of shifting to dairy alternatives (Figure 3b). Respondents who regularly consume meat have a lower intention to shift to meat alternatives. Similarly, respondents who consume large portion sizes are less likely to intend to shift their diet from meat or dairy to meat alternatives or dairy alternatives compared to respondents who consume small portion sizes. Respondents that received higher education are more likely to shift their consumption from meat to meat alternatives compared to respondents that did not receive higher education, especially in NLD. However, this effect was less obvious for dairy products. Additionally, younger respondents (18–33) were more likely to shift their consumption to both meat alternatives and dairy alternatives compared to older respondents (50–65). Moreover, people living in urban areas showed a higher preference to reduce meat consumption compared to those in rural areas.

**Figure 3:**
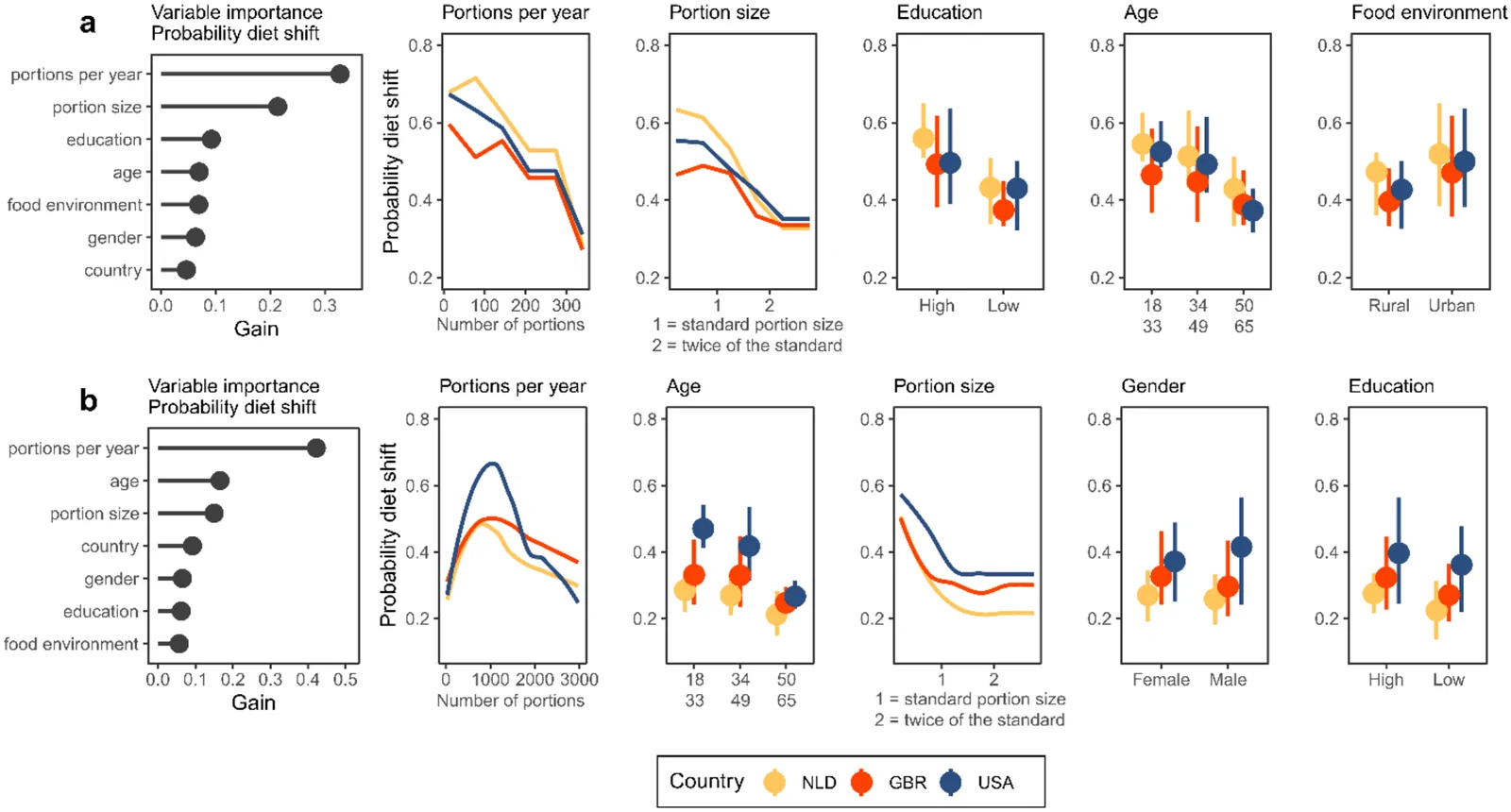
Probability of dietary shifts. (a), Meat and meat alternatives. The leftmost panel depicts the importance of the variables include in the model, where gain describes the increase in predictive power if the variable is included in the model. The following five panels show the response plots for the five most important variables grouped by country. (b), Dairy and dairy alternatives. The response plots are ordered by variable importance for meat and meat alternatives (a) and dairy and dairy alternatives (b). The error bars represent the 90% percentile interval derived from bootstrap resampling. If portion size is 2, it means the respondent consumed twice the amount of the standard portion size.

We further assessed the magnitude of potential biodiversity footprint reductions (i.e., Delta MSA-loss, %) among respondents intending to reduce their meat or dairy intake (Figure 4). Again, current consumption portions (meat/dairy portions per year) and portion size remained the most significant predictors of biodiversity footprint change. This means that respondents who consumed a higher number of meat portions per year as well as larger portion sizes, would generally exhibit smaller reductions in biodiversity footprint with their intended dietary changes. Respondents who had already reduced their meat consumption would show greater potential for biodiversity footprint reductions compared to those who expressed an intention to decrease their meat intake in the future. Female respondents demonstrated a higher reduction potential than males for meat products, while smaller differences were observed for dairy products. Age had a more pronounced influence on the reduction in biodiversity footprint for dairy products than for meat products. The XGBoost models based on RSR-loss showed similar patterns to those based on MSA-loss (Figure S8 and Figure S9).

**Figure 4:**
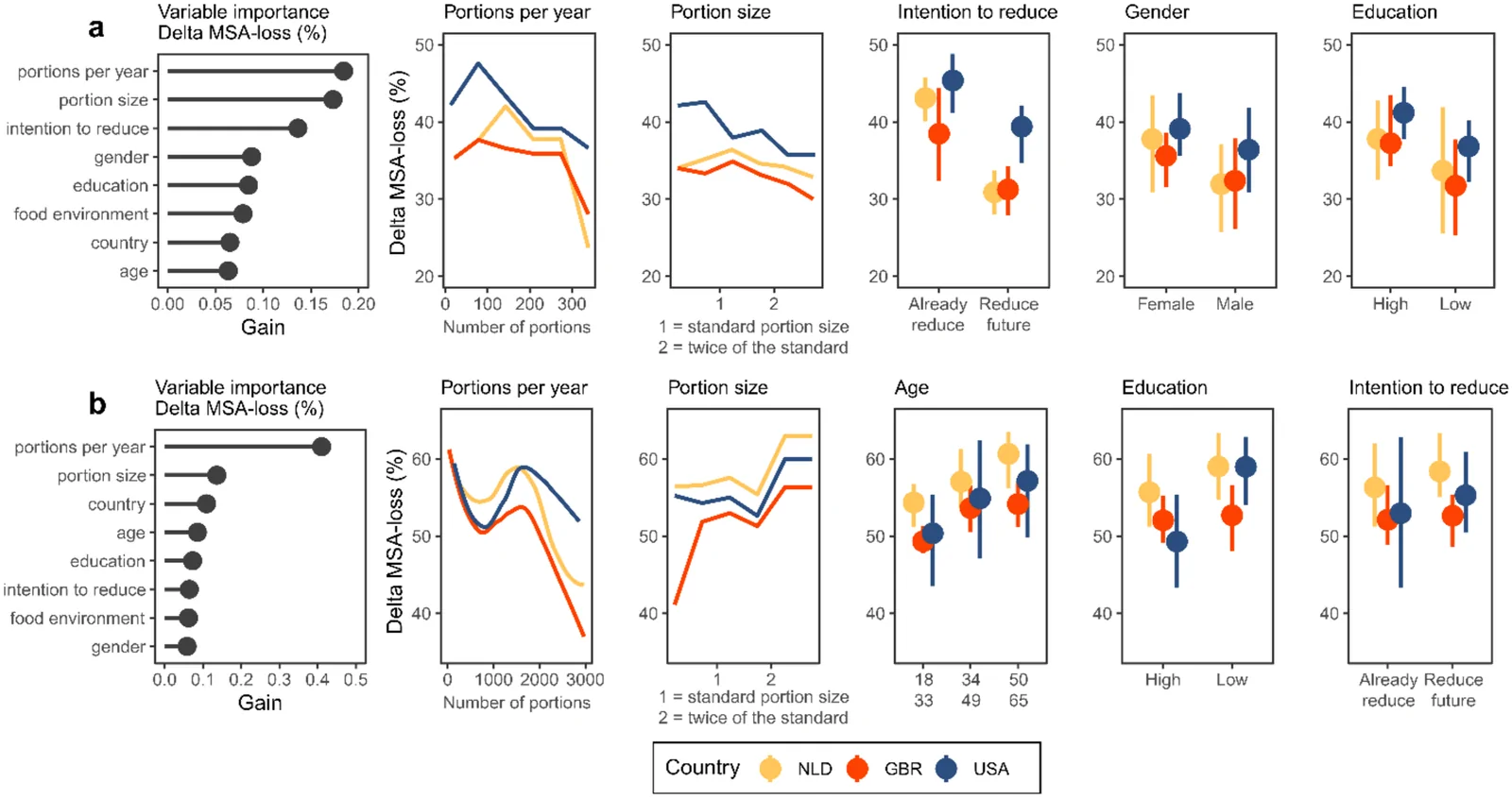
Prediction of delta MSA-loss (%) for respondents who intend to reduce their meat or dairy intake. (a), Meat and meat alternatives. The leftmost panel depicts the importance of the variables included in the model, where gain describes the increase in predictive power if the variable is included in the model. The following five panels show the response plots for the five most important variables grouped by country. (b), Dairy and dairy alternatives. The response plots are ordered by variable importance for meat and meat alternatives (a) and dairy and dairy alternatives (b). The error bars represent the 90% percentile interval derived from bootstrap resampling.

## Discussion

This study examined the biodiversity footprint associated with current and near future consumption of animal meat and meat alternatives (during main meals), dairy and dairy alternatives across respondents from the NLD, GBR and USA. Our findings demonstrate that diet-related biodiversity footprints vary substantially both within and between countries, reflecting the diversity in dietary habits and preferences among a large number of respondents. More than half of the respondents expressed no intention to reduce their consumption of animal meat and dairy products, suggesting that effective promotion of plant-based alternatives to meat and dairy remains a considerable challenge. Among those who did express intention to reduce meat or dairy intake, we found a large variability in potential biodiversity footprint reductions in the near future.

Around 40% of respondents in the NLD, GBR, and USA intend to reduce their meat or dairy intake, highlighting a significant mismatch between assumption-driven dietary scenarios and survey-based data. This emphasizes the need for more realistic approaches that take actual consumer behavior when estimating biodiversity benefits from dietary transitions than currently adopted in existing scenario studies.^14,27–29^ For instance, Kozicka et al.^30^ have quantified the potential climate and biodiversity impacts by assuming reductions of 10%, 25%, 50% and 90% in animal product consumption, replacing them with plant-based alternatives. These studies likely overestimate the potential for dietary change because they do not account for the substantial proportion of people who have no intention to reduce their meat and dairy intake. By assuming widespread adoption of plant-based diets, these models risk underestimating the challenges associated with promoting dietary shift on a large scale.

In addition, our results demonstrate that among respondents who intend to reduce meat or dairy consumption, the biodiversity footprint associated with their intended consumption patterns would significantly lower than that of their current intake, indicating the potential for considerable biodiversity loss mitigation. We also found that portion sizes play an important role in the biodiversity footprint of food consumption. This finding aligns with previous research emphasizing the role of portion size in shaping dietary impact. For instance, a study in the United Kingdom found that reducing the size of meat portions contributed the most to the overall decline in meat consumption, followed by other behavioral change strategies such as fewer meat-eating days.^31^ Given that we identified the number of annual meat (main meals) and dairy portions and portion size as key predictors of dietary shift probability and biodiversity footprint changes, our findings suggest that initiatives focusing on portion size reduction could be an effective strategy to lower the overall biodiversity footprint, particularly for respondents less inclined to adopt meat alternatives.

Moreover, our results reinforce the substantial contribution of animal products, particularly beef and lamb, to biodiversity loss, aligning with existing literature.^32–36^ By disaggregating broad meat categories into specific meat products, we captured significant variability in the respondents’ intent to decrease consumption of different types of meat products. For example, respondents were more likely to reduce beef consumption than poultry, revealing a level of granularity overlooked in prior research that focused on a general meat category.^37^ This enables a more accurate estimation of biodiversity footprint reductions associated with different dietary transitions. Interestingly, the pattern of reduction observed in individual food consumption aligns with broader dietary trends identified in previous studies.^38^ For example, our results show that the NLD consumes a higher proportion of plant-based foods compared to the GBR and USA, and younger people are more likely to have the intention to reduce meat and dairy intake than older generations. We found notable difference between intentions to shift from dairy to dairy alternatives. For example, few intended to replace cheese with plant-based cheese, possibly due to lower market availability and consumer adoption. This highlights the importance of targeting specific products within meat or dairy categories to maximize the biodiversity benefits through dietary shift.^39,40^

The biodiversity footprint intensity of many meat alternatives is lower than that of meat products, indicating that replacing certain animal-based products with alternatives could generate biodiversity benefits. Such benefits are particularly relevant in the context of national commitments to halt biodiversity loss, as in the NLD, GBR, and USA.^41–43^ However, this is not always the case across all alternative products. While plant-based alternatives generally reduced biodiversity footprints, the RSR-loss results showed that replacing dairy products with dairy alternatives was associated with an increase in biodiversity footprints in the USA. This was largely driven by plant-based desserts and plant-based ice cream containing coconut-derived ingredients, which were linked to sourcing regions such as Philippines, where RSR-loss characterization factors are relatively high, reflecting high species richness and endemism. These findings suggest that biodiversity outcomes depend not only on the replacement of animal-based products, but also on the composition and sourcing of the alternative products themselves. Shifting from animal-based to more plant-based diets could make a meaningful contribution toward achieving national biodiversity targets. However, intentions to reduce animal meat consumption vary substantially among individuals and across countries, a factor for consideration for the development of effective dietary policy frameworks. Survey responses on barriers to reducing meat and dairy products further highlight why these intentions may not readily translate into action. In particular, the most frequently reported barriers were the enjoyment of meat taste (∼72%) and texture (∼62%), whereas only about 40% of respondents associated reducing meat consumption with being “good for the planet”. Similarly, for dairy products, only 29% of respondents made such a link. These findings suggest that while sensory preferences remain strong, awareness of the environmental impacts of meat and dairy consumption remains limited.

One promising approach to promoting dietary shift is to implement population-level interventions that increase awareness of the link between animal product consumption and biodiversity loss. For example, integrating biodiversity considerations into national food-based dietary guidelines would offer a concrete step toward environmental objectives.^27^ In addition, guidelines could specify recommended portion sizes for animal products or suggest plant-based alternatives, potentially helping people make more sustainable choices.^3,31^ However, their effectiveness would depend on consumers’ willingness to adjust their current eating habits, which remains limited as our findings suggest. Beyond dietary guidelines, broader intervention strategies targeting education, public communication, and restructuring of food environments—for instance, through enhanced access to plant-based alternatives and portion size adjustments—can promote dietary shifts. Recent evidence has shown that interventions combining education and communication-based strategies tend to be the most effective in promoting sustainable diet transitions, highlighting the need for coordinated, multilevel action to translate individual intentions into large-scale biodiversity benefits.^44^

Although our analysis offers insights into the biodiversity loss mitigation of realistic shifts in consumer diets, it is subject to several limitations and model uncertainties. First, our study assessed the potential reduction in biodiversity footprint based on self-reported near future dietary intentions obtained from survey responses. Intentions are an important component of many behavior change models,^45–47^ and previous research has shown that stronger intentions are associated with greater reductions in meat consumption^48,49^ and a higher willingness to pay for plant-based alternatives.^50^ However, intentions do not necessarily translate into actual behavior change,^51,52^ because dietary behavior is often habitual and influenced by taste preferences, availability of alternatives and affordability. Consequently, the estimated biodiversity footprint reductions should be regarded as an upper-bound estimate of what could be achieved if these intentions result in sustained dietary change. To better account for variability in dietary change, we distinguished respondents into different stages of dietary change, including those who had already reduced meat or dairy consumption, those who had not yet but plan to reduce meat or dairy consumption within the next six months and those who do not intend to change their diet. Focusing on those who have reduced or express an intention to reduce meat or dairy consumption provides a more realistic basis for estimating the potential biodiversity footprint reductions associated with dietary change compared to hypothetical dietary scenarios. Nevertheless, the actual magnitude of biodiversity footprint reductions will ultimately depend on the extent to which intended dietary changes are adopted and maintained over time. Second, our focus was limited to meat and meat alternatives consumed during the main meal, as well as dairy and dairy alternatives consumed during the day. This does not capture the entire spectrum of dietary intake, indicating that contributions from other food groups (e.g., grains, beverages) were not included. Although meat products contribute the most to biodiversity footprints, this scope limits our ability to assess total dietary biodiversity impacts. In addition, seafood (including both aquaculture and wild-caught fish) was not included in our analysis due to the lack of data and methods to quantify biodiversity footprints of seafood consumption,^19^ even though survey respondents indicated potential shifts in that direction. As a result, the biodiversity footprints related to reducing and replacing meat with seafood were not assessed. Third, the self-reported dietary intakes differ from national FAO food consumption statistics. FAO estimates are derived from food balance sheets that account for domestic production and trade and represent annual food availability at the population level, whereas our survey captured participants’ typical daily consumption of meat, dairy, and alternative products in their main meal (which may under- or overestimate annual average consumption). However, previous studies comparing dietary surveys with FAO statistics have found that FAO estimates can substantially exceed reported consumption of meat and milk.^53^ This discrepancy may arise because FAO food balance sheets do not fully account for losses during processing, retail, and final consumption and may include food not intended for human consumption such as pet food.^54^ Consumption surveys therefore provide valuable information on individual dietary intake and intended dietary shifts that cannot be captured by food balance sheets. Fourth, biodiversity footprint estimates are subject to uncertainties related to the representation of agricultural management practices and livestock production systems. Empirical research has demonstrated that biodiversity responses vary across pasture management intensities,^55^ yet current footprint methods do not adequately distinguish management systems, such as extensive rangeland versus intensive livestock production.^56^ As a result, footprint estimates do not fully capture the variability in biodiversity impacts associated with different production practices. To reduce uncertainty in biodiversity footprints, we therefore recommend the development of footprint methods that distinguish agricultural management and livestock production intensity as a promising avenue for future research. Another uncertainty concerns the allocation of inputs in multioutput processes. Previous research has shown that the choice between mass- and value-based allocation can substantially affect footprint estimates for livestock products, indicating that our results may be sensitive to the selected allocation method.^18^ We used the FABIO physical input-output table, which applies mass-based allocation to assign inputs in multioutput processes. Biophysical allocation is the most commonly used allocation approach and is recommended by life cycle assessment guidelines for livestock production,^57,58^ because value-based allocation depends on market prices, which vary across regions, production systems, and over time.^57,59^ Finally, our analysis focused exclusively on the NLD, GBR, and USA. While these countries may be representative of Western dietary patterns, they do not reflect the dietary habits of many other regions, such as Global South and East Asia, where both consumption patterns and biodiversity footprints may differ substantially.

In summary, substituting high biodiversity impact food products with plant-based alternatives offers a promising near-future opportunity to accelerate biodiversity loss mitigation. However, across all survey respondents in the NLD, GBR, and USA who expressed an intention to reduce meat consumption, the estimated future dietary changes would lead to an average biodiversity footprint reduction of only 13% when compared to their current meat and meat alternative intake. Relying solely on consumer motivation is therefore unlikely to be sufficient to achieve substantial biodiversity loss mitigation via dietary change. This suggests that realizing biodiversity footprint reductions through dietary shift remains a considerable challenge. It is essential to consider actual eating behaviors and perceived barriers to change to enable effective dietary transitions.

## Supplementary Material





## Notes

Tables provide additional information on portion size, food product mapping, and country ISO3 codes (Tables S1–S3); figures provide additional results supporting the analysis (Figures S1–S9) (PDF)
Text provides information on the consumer survey and methodological details for the quantifications (Text Sections S1–S5) (PDF)
