# Supplementary material for: Potential Biodiversity Footprint Reductions from Intended Shifts to Plant-Based Meals
Source: Environ Sci Technol. 2026 Sep 24;60(39):27743–53. doi: 10.1021/acs.est.6c02675 (PMC13648232; doi:10.1021/acs.est.6c02675)
Supplement: Supplementary file 1 [file es6c02675_si_001.pdf]

SI – Supplementary Information

Potential biodiversity footprint reductions from intended shifts to plant-based meals

Hanzhong Zheng[a*], *Koen J. J. Kuipers*[a], *Selwyn Hoeks*[a], *Mark A. J. Huijbregts*[a], *Nicole Neufingerl*[b], *Elizabeth H. Zandstra*[b,c], *Sarah Sim*[a,d]

[a] Department of Environmental Science, Radboud Institute for Biological and Environmental Sciences, Radboud University, Heyendaalseweg 135, Nijmegen 6525AJ, The Netherlands

[b] Unilever Foods Innovation Centre Wageningen, Bronland 14, Wageningen 6708WH, The Netherlands

[c] Division of human Nutrition and Health, Wageningen University & Research, Stippeneng 4, Wageningen 6708WH, The Netherlands

[d] Safety, Environmental and Regulatory Sciences, Unilever R&D, Colworth Science Park, Sharnbrook, Bedfordshire MK44 1LQ, United Kingdom

**Corresponding author*: Hanzhong Zheng (hanzhong.zheng@ru.nl)

This SI file including 18 pages, 3 tables, and 9 figures.

Text Contents

Text Section S1. Survey……S3
Text Section S2. Survey population sample……S3
Text Section S3. Stages of change……S3
Text Section S4. Transform frequency into yearly average number of portions……S4
Text Section S5. Conversion factors link food product to primary agricultural commodity..S4

Table Contents

Table S1. Standard portion size assumed for food products in the survey……S5
Table S2. The food product used in the survey and linkage with FABIO……S6
Table S3. Country information for FABIO……S7

Figure Contents

Fig.S1. The biodiversity footprint (RSR-loss) related to average current intake of meat, meat alternatives, dairy, and dairy alternatives per person per year……S10
Fig.S2. Average portion size and percentage contribution of respondents in different states of change to biodiversity footprint……S11
Fig.S3. Relative change in biodiversity footprint (MSA-loss) of meat, dairy, and alternatives compared to current diet……S12
Fig.S4. Relative change in biodiversity footprint (RSR-loss) of meat, dairy, and alternatives compared to current diet……S13
Fig.S5. Change in MSA-loss associated with intended dietary shifts by sourcing countriesS14
Fig.S6. Dietary shift in the near future (RSR-loss)……S15
Fig.S7. Change in RSR-loss associated with intended dietary shifts by sourcing countries.S16
Fig.S8. Probability of dietary shifts for RSR-loss……S17
Fig.S9. Prediction of delta RSR-loss (%) for respondents who intend to reduce their meat or dairy intake……S18

Text Section S1. Survey Please see separate *Survey* PDF.

Text Section S2. Survey population sample

To obtain diverse and broadly comparable samples across countries, soft quota sampling was applied in the recruitment of respondents. Quotas were set for gender, age, education level, and food environment for urban/rural. Per country, we aimed for approximately equal distributions across key sociodemographic strata, including gender (male and female); three age groups (18-33, 34-49, 50-65 years); and low versus high education levels, defined as having obtained at least university (i.e., undergraduate) degree or not. In addition, respondents were sampled to achieve a balance between urban and rural areas.

Text Section S3. Stages of change

We assessed the respondents' (self-reported) interest in engaging in reducing meat consumption and dairy consumption (i.e., which stage of behavioural change they were in), based on the five stages of the Trans Theoretical Model of Behavioural Change (Prochaska & Velicer, 1997). Respondents were asked to describe which statement best represented their current interest in reducing meat and dairy among the following options:

Stage	Description	Group
Stage 1	I am not at all interested in reducing meat / dairy in my diet and I have no intention in doing so in the next 6 months	No intention to reduce (Stage 1)
Stage 2	I am interested in reducing meat / dairy and I have the intention of doing that within the next 6 months	Intended & willing to reduce (Stage 2 and 3)
Stage 3	I am interested in reducing meat / dairy in my diet and I have the intention in doing that in the next month	
Stage 4	I am interested in reducing meat /dairy in my diet and I have started reducing meat / dairy during the last the last 6 months	Already reducing (Stage 4 and 5).
Stage 5	I am interested in reducing meat / dairy in my diet and I have already reduced meat / dairy in my diet for longer than 6 months	

Prochaska, J.O.; Velicer, W.F. The Transtheoretical Model of Health Behavior Change. *Am. J. Health Promot.* **1997**, 12, 38–48.

Text Section S4. Transform frequency into yearly average number of portions

To estimate the yearly average number of meat portions based on respondents' reported weekly consumption frequency, we apply the following transformation method. Each frequency category is converted into an equivalent annual consumption rate by determining the proportional weekly intake and extrapolating it over a full year (365 days).

For instance, if a respondent selects ‘once per month’ we approximate monthly consumption as (1/30) portions per day. Multiplying by 365 days, this results in an estimated 12.17= [(1/30)*365] portions per year. Similarly, for those who consume meat ‘4-5 times per week’, we take the midpoint (4.5 times per week) and convert it into a daily rate (4.5/7 portions per day). Multiplying this by 365 days yields an estimated 234.64 = [(4.5/7)*365] portions per year. This approach allows for a standardized estimation of annual meat consumption across varying reported frequencies.

Text Section S5. Conversion factors link food product to primary agricultural commodity

To assess the biodiversity footprint of the 22 food products covered in the survey, we first mapped these food products to primary agricultural commodities in FABIO. Of the 22 food products we identified 12 single ingredient foods (e.g., beef, vegetable) that could be directly linked to primary commodities in FABIO, and 10 processed foods (e.g., tofu) which we matched with commodities in FABIO by using their key raw ingredients (e.g., linking tofu to soybean) (see Table S2). We obtained the raw ingredients of food products from the Open Food Facts database. We applied conversion factors to estimate the quantity of primary agricultural commodities (in kg) required for the final consumed products. We applied conversion factors considering water content in cooked or processed items (e.g., cooked beef) to estimate the demand for raw product, based on USDA Agricultural Research Service data (USDA, 2018). These factors were calculated by comparing the water content of a 100g of a raw item with the water content of 100g of a processed item. We also applied conversion factors to estimate the amount of raw soybean required for producing soybean derivatives (e.g., plant-based meat). Finally, we applied conversion factors for the amount of required milk to produce milk-based products (e.g., cheese).

Open Food Facts. https://world.openfoodfacts.org/

USDA. FoodData Central. U.S. Department of Agriculture. 2018 https://fdc.nal.usda.gov/

Table S1. Standard portion size assumed for food products in the survey

Section	Food Category	No.	Food product	Abbreviation	Suggested portion (g)
Meat	Animal meat	1	Beef	Beef	100
		2	Poultry	Poultry	100
		3	Pork	Pork	100
		4	Lamb	Lamb	100
	(Plant-based) meat alternatives	5	Fish & Seafood	Seafood	120
		6	Eggs	Eggs	50
		7	Cheese and dairy items	CheeseItems	100
		8	Plant-based alternative mimicking the taste and texture of meat	Pb_meat (mimic meat)	100
		9	Pulses	Pulses	135
		10	Tofu & tempeh	Tofu	85
		11	Nuts & seeds	Nuts	30
		12	Vegetables	Vegetables	80
Dairy	Dairy	13	Milk & dairy drinks	Milk	250
		14	Yoghurt or quark	Yoghurt	150
		15	Ice cream	Ice cream	100
		16	Dairy-Dessert	Dessert	150
		17	Cheese	Cheese	30
	Plant-based dairy alternatives	18	Plant-based milk	Pb_milk	250
		19	Plant-based yoghurt	Pb_yoghurt	150
		20	Plant-based ice cream	Pb_icecream	100
		21	Plant-based dessert	Pb_dessert	150
		22	Plant-based cheese	Pb_cheese	30

Note: The abbreviation column serves as the legend for all figures included in the main text.

Table S2. The food product used in the survey and linkage with FABIO

Section	Food category	No	Food product	Type	Link FABIO
Meat	Animal meat	1	Beef	S	Bovine meat
		2	Poultry	S	Poultry meat
		3	Pork	S	Pigmeat
		4	Lamb	S	Mutton and goat meat
	(Plant-based) meat alternatives	5	Fish & Seafood	S	Fish seafood
		6	Eggs	S	Eggs
		7	Cheese and dairy items	P	Milk, wheat and products, vegetable oil, rapeseed oil
		8	Plant-based alternative mimicking the taste and texture of meat	P	Beans, peas, pulses other, egg, sunflower oil, wheat and product
		9	Pulses	S	Beans, peas, pulses other
		10	Tofu & tempeh	P	Soyabeans
		11	Nuts & seeds	S	Nuts and products
		12	Vegetables	S	Tomatoes, onions, vegetables other
Dairy	Dairy	13	Milk & dairy drinks	S	Milk
		14	Yoghurt or quark	S	Milk
		15	Ice cream	P	Sugar, egg, milk, soyabeans
		16	Dairy Dessert	P	Milk
		17	Cheese	S	Milk
	Plant-based dairy alternatives	18	Plant-based milk	P	Soyabeans<br>Nuts and products
		19	Plant-based yoghurt	P	Soyabeans, coconuts
		20	Plant-based ice cream	P	Soyabeans, coconuts
		21	Plant-based dessert	P	Soyabeans, coconuts
		22	Plant-based cheese	P	Potatoes and products, coconuts oil

Note: S is single ingredient food; P is processed food.

Table S3. Country information for FABIO

Area_code	iso3c	Area	Area_code	iso3c	Area
1	ARM	Armenia	52	AZE	Azerbaijan
2	AFG	Afghanistan	53	BEN	Benin
3	ALB	Albania	54	DNK	Denmark
4	DZA	Algeria	55	DMA	Dominica
7	AGO	Angola	56	DOM	Dominican Republic
8	ATG	Antigua and Barbuda	57	BLR	Belarus
9	ARG	Argentina	58	ECU	Ecuador
10	AUS	Australia	59	EGY	Egypt
11	AUT	Austria	60	SLV	El Salvador
12	BHS	Bahamas	63	EST	Estonia
13	BHR	Bahrain	66	FJI	Fiji
14	BRB	Barbados	67	FIN	Finland
15	BLX	Belgium-Luxembourg	68	FRA	France
16	BGD	Bangladesh	70	PYF	French Polynesia
19	BOL	Bolivia	72	DJI	Djibouti
20	BWA	Botswana	73	GEO	Georgia
21	BRA	Brazil	74	GAB	Gabon
23	BLZ	Belize	75	GMB	Gambia
25	SLB	Solomon Islands	79	DEU	Germany
26	BRN	Brunei Darussalam	80	BIH	Bosnia and Herzegovina
27	BGR	Bulgaria	81	GHA	Ghana
28	MMR	Myanmar	83	KIR	Kiribati
29	BDI	Burundi	84	GRC	Greece
32	CMR	Cameroon	86	GRD	Grenada
33	CAN	Canada	89	GTM	Guatemala
35	CPV	Cabo Verde	90	GIN	Guinea
37	CAF	Central African Republic	91	GUY	Guyana
38	LKA	Sri Lanka	93	HTI	Haiti
39	TCD	Chad	95	HND	Honduras
40	CHL	Chile	96	HKG	China, Hong Kong SAR
41	CHN	China, mainland	97	HUN	Hungary
44	COL	Colombia	98	HRV	Croatia
46	COG	Congo	99	ISL	Iceland
48	CRI	Costa Rica	100	IND	India
49	CUB	Cuba	101	IDN	Indonesia
50	CYP	Cyprus	102	IRN	Iran (Islamic Republic of)
51	CSK	Czechoslovakia	103	IRQ	Iraq

Area_code	iso3c	Area	Area_code	iso3c	Area
104	IRL	Ireland	149	NPL	Nepal
105	ISR	Israel	150	NLD	Netherlands
106	ITA	Italy	151	ANT	Netherlands Antilles
107	CIV	Côte d'Ivoire	153	NCL	New Caledonia
108	KAZ	Kazakhstan	154	MKD	North Macedonia
109	JAM	Jamaica	155	VUT	Vanuatu
110	JPN	Japan	156	NZL	New Zealand
112	JOR	Jordan	157	NIC	Nicaragua
113	KGZ	Kyrgyzstan	158	NER	Niger
114	KEN	Kenya	159	NGA	Nigeria
115	KHM	Cambodia	162	NOR	Norway
116	PRK	Democratic People's Republic of Korea	165	PAK	Pakistan
117	KOR	Republic of Korea	166	PAN	Panama
118	KWT	Kuwait	167	CZE	Czech Republic
119	LVA	Latvia	168	PNG	Papua New Guinea
120	LAO	Lao People's Democratic Republic	169	PRY	Paraguay
121	LBN	Lebanon	170	PER	Peru
122	LSO	Lesotho	171	PHL	Philippines
123	LBR	Liberia	173	POL	Poland
124	LBY	Libya	174	PRT	Portugal
126	LTU	Lithuania	175	GNB	Guinea-Bissau
128	MAC	China, Macao SAR	176	TLS	Timor-Leste
129	MDG	Madagascar	177	PRI	Puerto Rico
130	MWI	Malawi	178	ERI	Eritrea
131	MYS	Malaysia	179	QAT	Qatar
132	MDV	Maldives	181	ZWE	Zimbabwe
133	MLI	Mali	183	ROU	Romania
134	MLT	Malta	184	RWA	Rwanda
136	MRT	Mauritania	185	RUS	Russian Federation
137	MUS	Mauritius	186	SCG	Serbia and Montenegro
138	MEX	Mexico	188	KNA	Saint Kitts and Nevis
141	MNG	Mongolia	189	LCA	Saint Lucia
143	MAR	Morocco	191	VCT	Saint Vincent and the Grenadines
144	MOZ	Mozambique	193	STP	Sao Tome and Principe
146	MDA	Republic of Moldova	194	SAU	Saudi Arabia
147	NAM	Namibia	195	SEN	Senegal

Area_code	iso3c	Area	Area_code	iso3c	Area
197	SLE	Sierra Leone	226	UGA	Uganda
198	SVN	Slovenia	228	SUN	USSR
199	SVK	Slovakia	229	GBR	United Kingdom
200	SGP	Singapore	230	UKR	Ukraine
201	SOM	Somalia	231	USA	United States of America
202	ZAF	South Africa	233	BFA	Burkina Faso
203	ESP	Spain	234	URY	Uruguay
207	SUR	Suriname	235	UZB	Uzbekistan
208	TJK	Tajikistan	236	VEN	Venezuela (Bolivarian Republic of)
209	SWZ	Eswatini	237	VNM	Viet Nam
210	SWE	Sweden	238	ETH	Ethiopia
211	CHE	Switzerland	244	WSM	Samoa
212	SYR	Syrian Arab Republic	248	YUG	Yugoslav SFR
213	TKM	Turkmenistan	249	YEM	Yemen
214	TWN	China, Taiwan Province of	250	COD	Democratic Republic of the Congo
215	TZA	United Republic of Tanzania	251	ZMB	Zambia
216	THA	Thailand	255	BEL	Belgium
217	TGO	Togo	256	LUX	Luxembourg
220	TTO	Trinidad and Tobago	272	SRB	Serbia
221	OMN	Oman	273	MNE	Montenegro
222	TUN	Tunisia	276	SDN	Sudan
223	TUR	Turkey	277	SSD	South Sudan
225	ARE	United Arab Emirates	999	ROW	RoW

Figure S1.

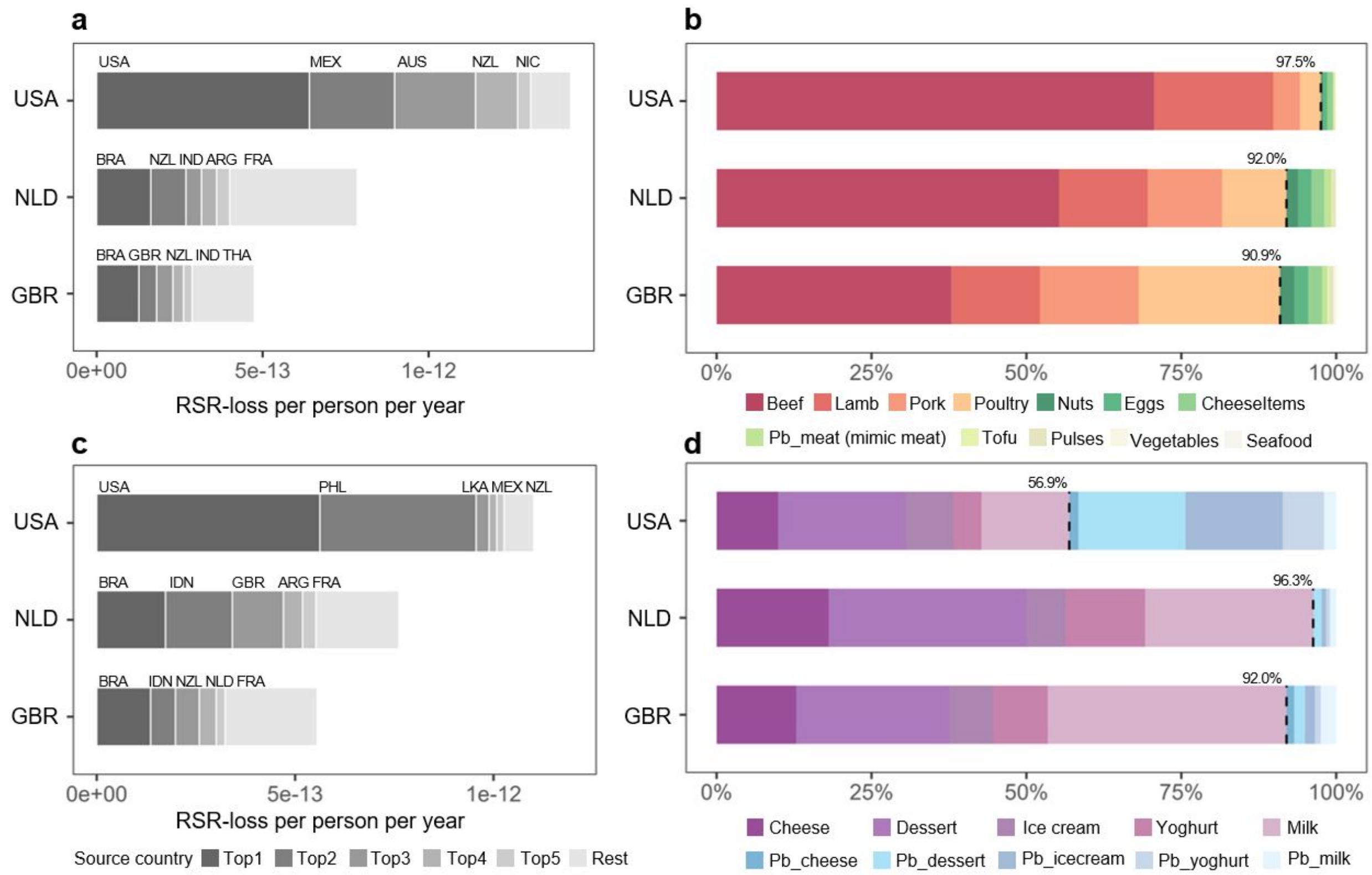

Fig. S1 | The biodiversity footprint (RSR-loss) related to average current intake of meat, meat alternatives, dairy, and dairy alternatives per person per year. **a**, The absolute RSR-loss of current consumption of meat and meat alternatives by sourcing countries. **b**, Contribution analysis of meat and meat alternatives to biodiversity footprint. **c**, The absolute RSR-loss of current consumption of dairy and dairy alternatives by sourcing countries. **d**, Contribution analysis of dairy and plant-based (Pb) dairy alternatives to biodiversity footprint. The sourcing countries in panels **a** and **c** show the 5 largest contributing countries, with all remaining countries aggregated as Rest. The iso3 country codes displayed above each bar indicate the top 5 sourcing countries in descending order. The dashed line in panels **b** and **d** presents the separator between meat/dairy and alternatives. Biodiversity footprints are estimated based on the average of the current intake across respondents.

Figure S2.

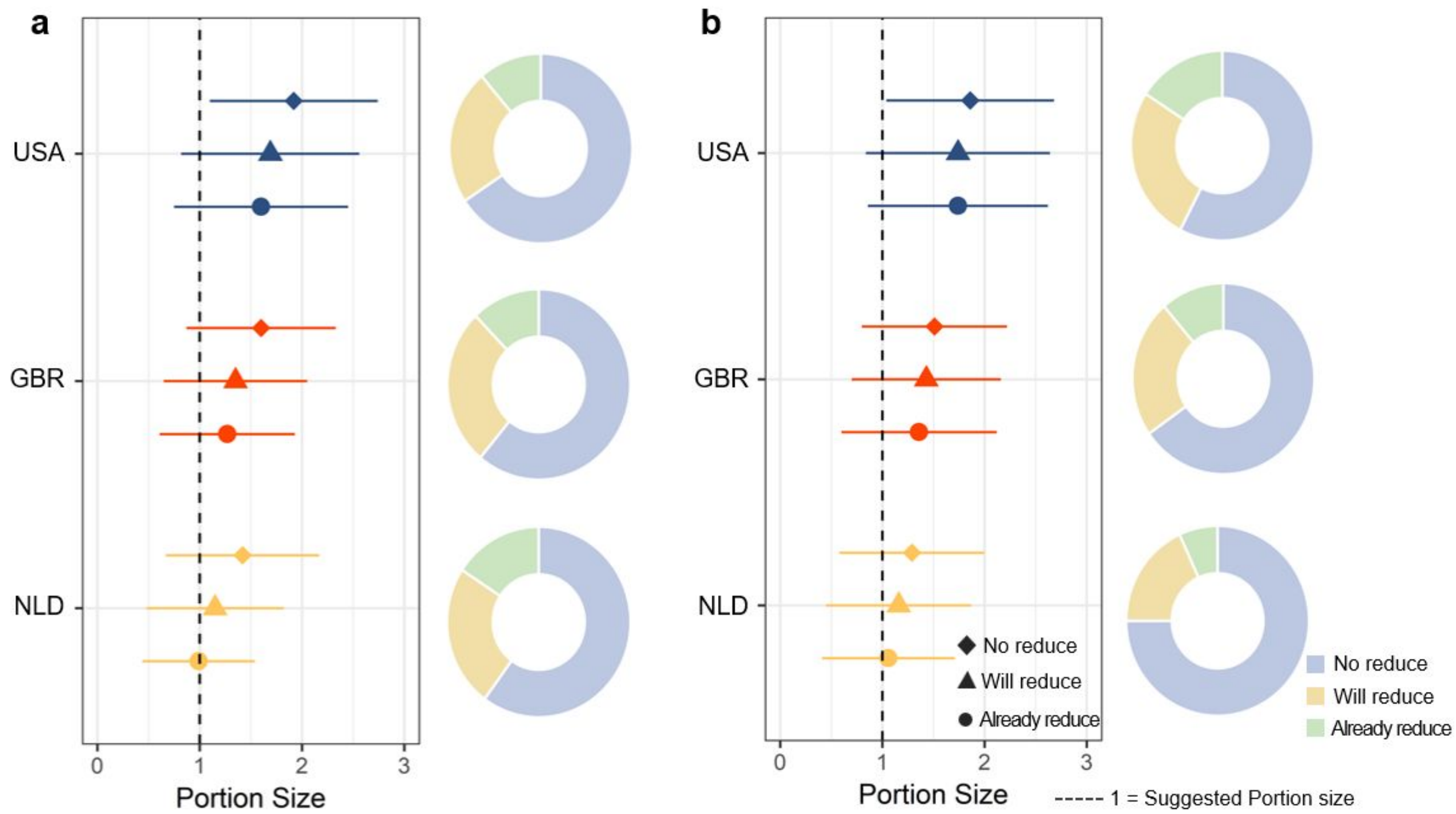

Fig. S2 | Average portion size and percentage contribution of respondents in different states of change to biodiversity footprint. **a**, Meat and meat alternatives. **b**, Dairy and dairy alternatives. The portion size expressed as a ratio (1=100%) of the standard portion size, for example, the largest portion size in the survey (277 g) equals to 2.77 here. We show the results as mean and standard deviation (mean $\pm$ s.d.). The donut chart shows the % contribution of each dietary behaviour group to the average total biodiversity footprint.

Figure S3.

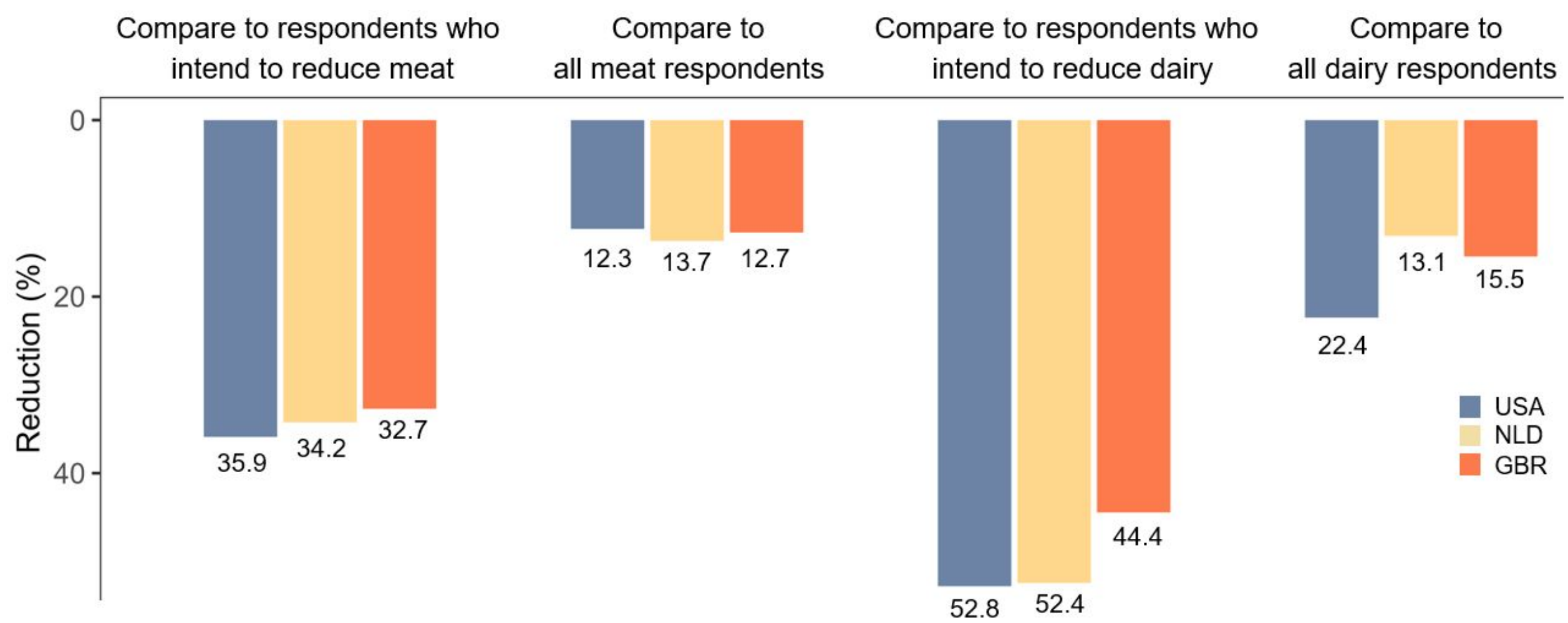

Fig. S3 | Relative change in biodiversity footprint (MSA-loss) of meat, meat alternatives, dairy, and dairy alternatives compared to current diet. The first panel shows the relative change compared to respondents who intend to reduce meat and meat alternatives. The second panel shows the relative change compared to all respondents regarding meat and meat alternatives. The third panel shows the relative change compared to those who intend to reduce dairy and dairy alternatives. The last panel shows the relative change compared to all respondents regarding dairy and dairy alternatives.

Figure S4.

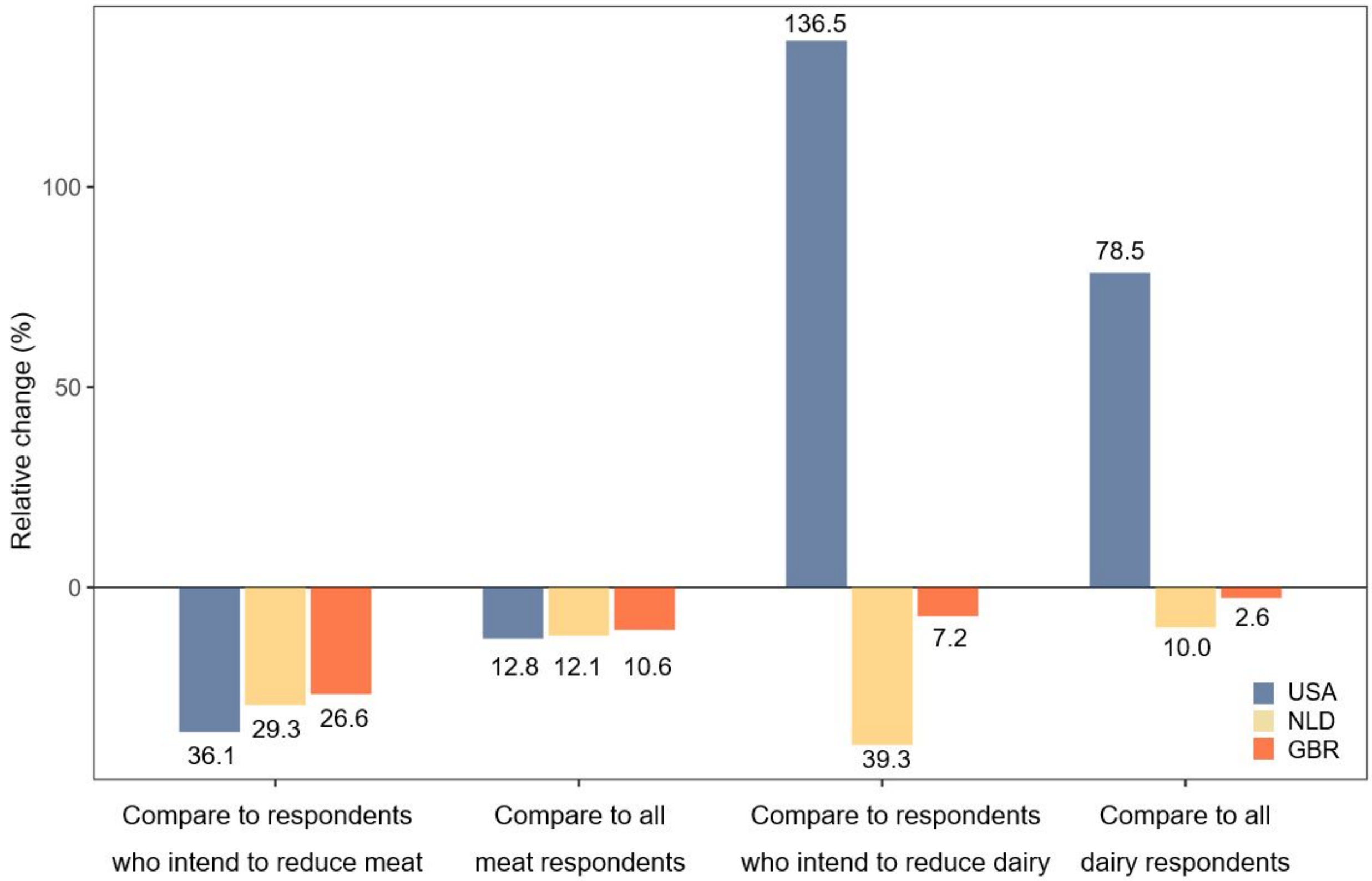

Fig. S4 | Relative change in biodiversity footprint (RSR-loss) of meat, meat alternatives, dairy, and dairy alternatives compared to current diet. The first panel shows the relative change compared to respondents who intend to reduce meat and meat alternatives. The second panel shows the relative change compared to all respondents regarding meat and meat alternatives. The third panel shows the relative change compared to those who intend to reduce dairy and dairy alternatives. The last panel shows the relative change compared to all respondents regarding dairy and dairy alternatives. Values below y=0 indicate reductions, whereas values above y=0 indicate increases.

Figure S5.

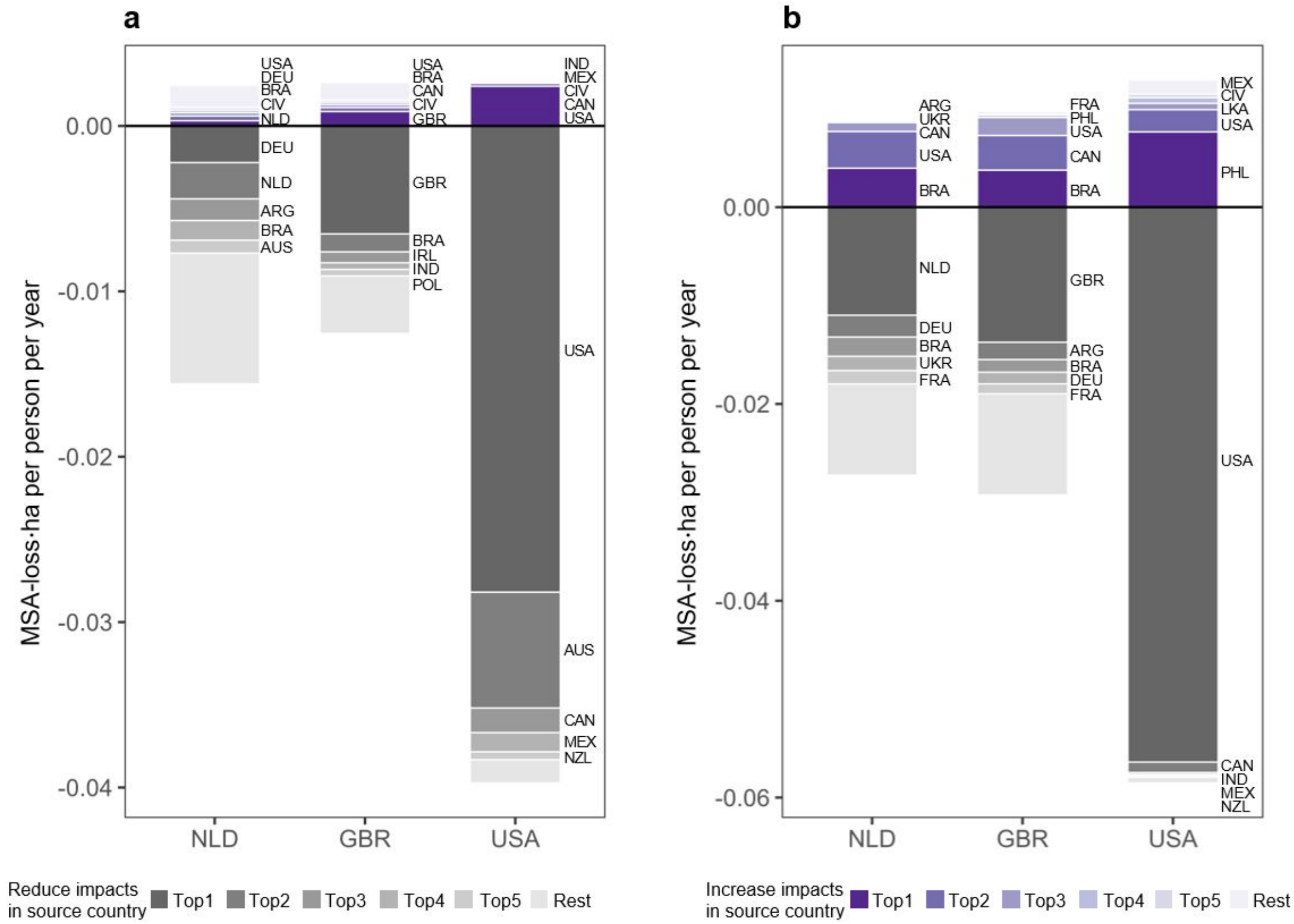

Fig. S5 | Change in MSA-loss associated with intended dietary shifts by sourcing countries. a, Replacement of meat with meat alternatives. **b**, Replacement of dairy with dairy alternatives. Grey bars show the 5 sourcing countries with the largest avoided biodiversity footprints, with all remaining countries aggregated as Rest. The iso3 country codes displayed next to each grey bar indicate the corresponding sourcing countries in descending order (from *y*=0 to bottom) of avoided footprints. Purple bars show the 5 sourcing countries contributing the largest increases in biodiversity footprints, with all remaining countries aggregated as Rest. The iso3 country codes displayed next to each purple bar indicate the corresponding sourcing countries in descending order (from *y*=0 to top) of increased footprints.

Figure S6.

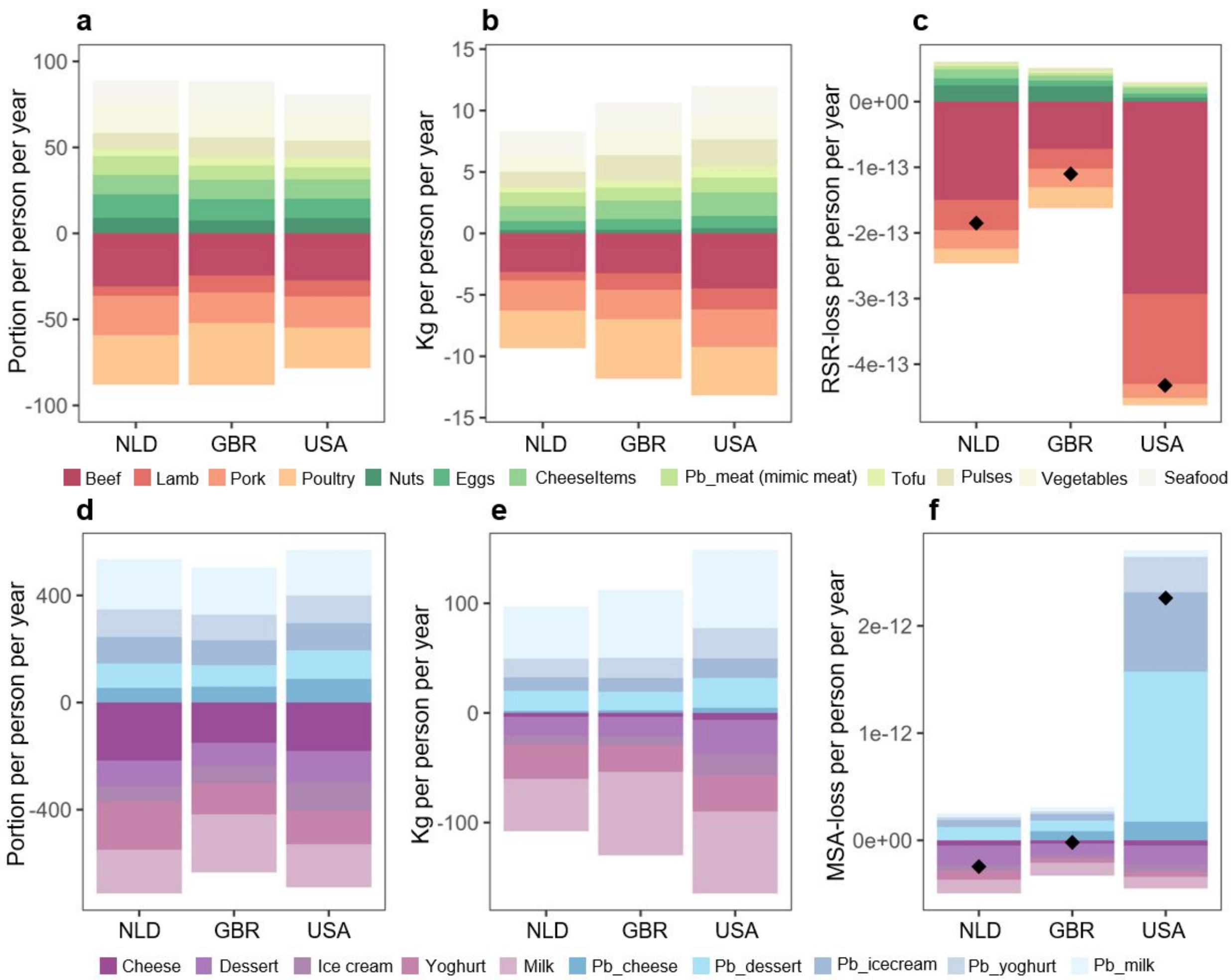

Fig. S6 | Dietary shift in the near future (RSR-loss). **a**,**b**,**c** Estimated reduction of meat consumption and increased consumption of meat alternatives expressed as portions per person y^{-1}(**a**), Kg per person y^{-1} (**b**), and related change in RSR-loss · ha per person y^{-1}(**c**) in terms to those people who indicate intention to reduce meat. **d,e,f**, Estimated reduction of dairy consumption and increased consumption of plant-based (Pb) dairy alternatives expressed as portions (**d**), Kg (**e**) and related change in RSR-loss (**f**) among those respondents who intend to reduce dairy intake. The portions, kg, and RSR-loss · ha are reported as the average across respondents. The diamond in the RSR-loss panel represents the net change (reduction) in RSR-loss · ha for each country.

Figure S7.

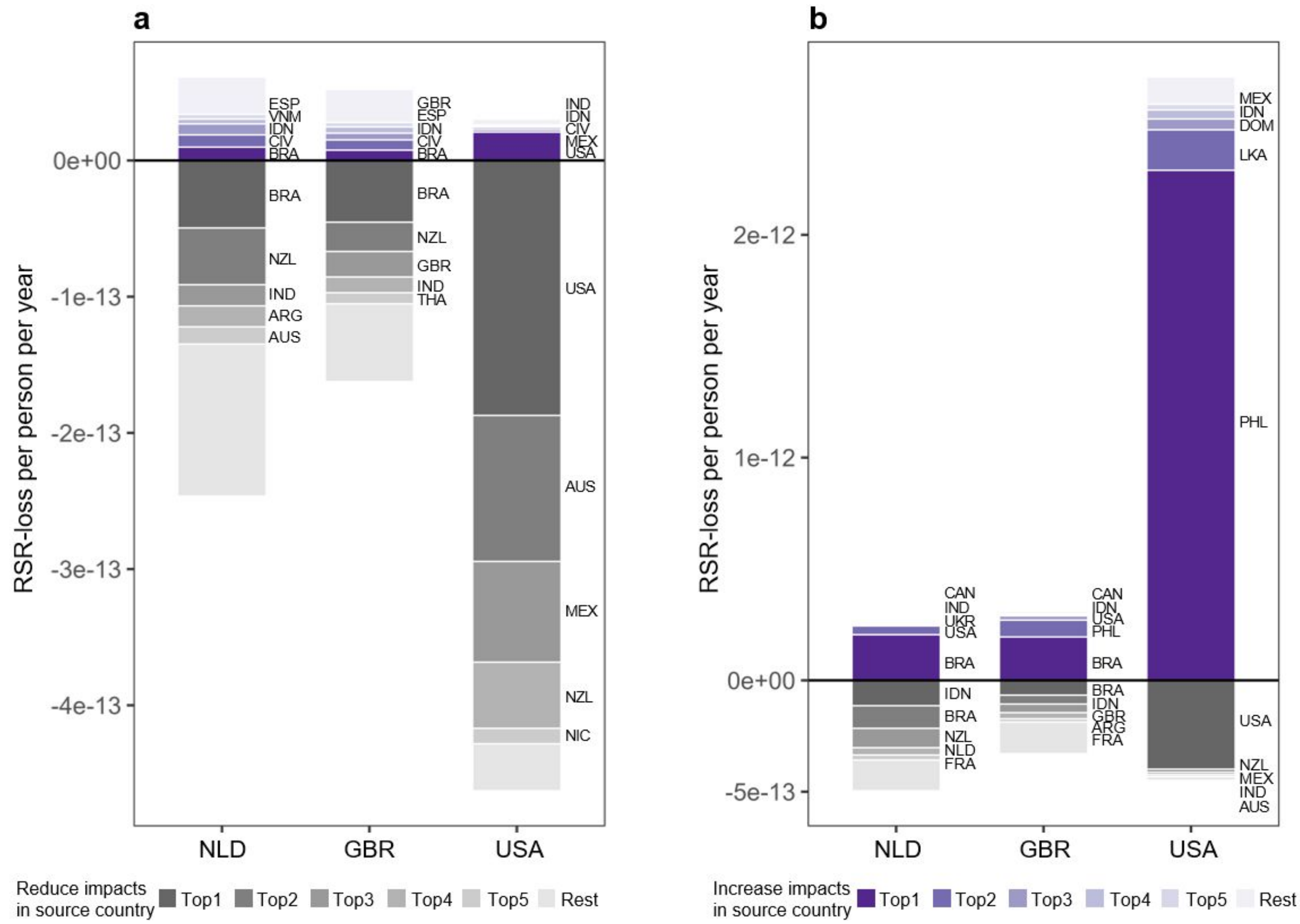

Fig. S7 | Change in RSR-loss associated with intended dietary shifts by sourcing countries. a, Replacement of meat with meat alternatives. **b**, Replacement of dairy with dairy alternatives. Grey bars show the 5 sourcing countries with the largest avoided biodiversity footprints, with all remaining countries aggregated as Rest. The iso3 country codes displayed next to each grey bar indicate the corresponding sourcing countries in descending order (from y=0 to bottom) of avoided footprints. Purple bars show the 5 sourcing countries contributing the largest increases in biodiversity footprints, with all remaining countries aggregated as Rest. The iso3 country codes displayed next to each purple bar indicate the corresponding sourcing countries in descending order (from y=0 to top) of increased footprints.

Figure S8.

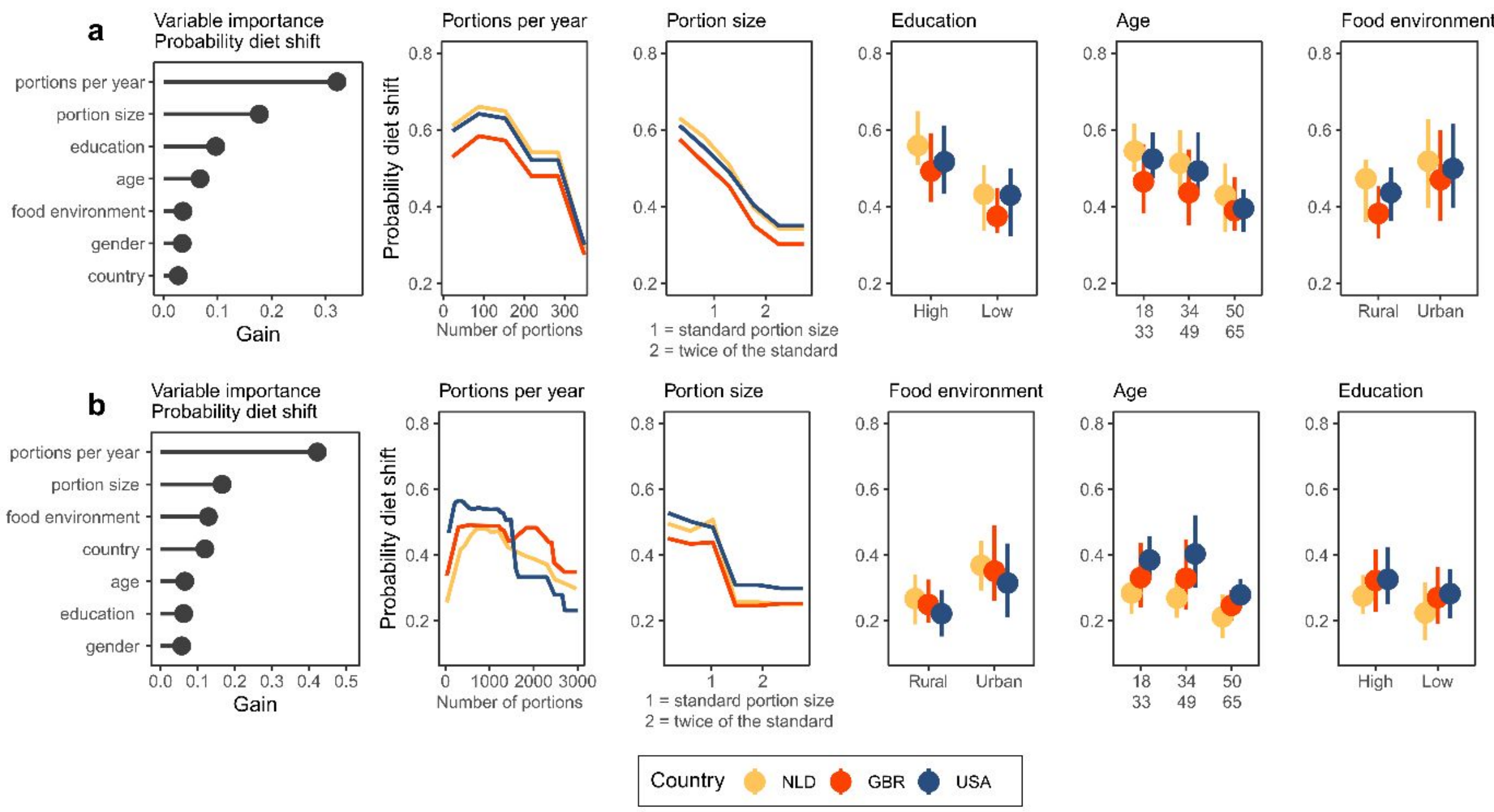

Fig. S8 | Probability of dietary shifts for RSR-loss. **a**, Meat and meat alternatives. The leftmost panel depicts the importance of the variables include in the model, where gain describes the increase in predictive power if the variable is included in the model. The following five panels show the response plots for the five most important variables grouped by country. **b**, Dairy and dairy alternatives. The response plots are ordered by variable importance for meat and meat alternatives (a) and dairy and dairy alternatives (b). The error bars represent the 90% percentile interval derived from bootstrap resampling. If portion size is 2, it means the respondent consumed twice the amount of the standard portion size.

Figure S9.

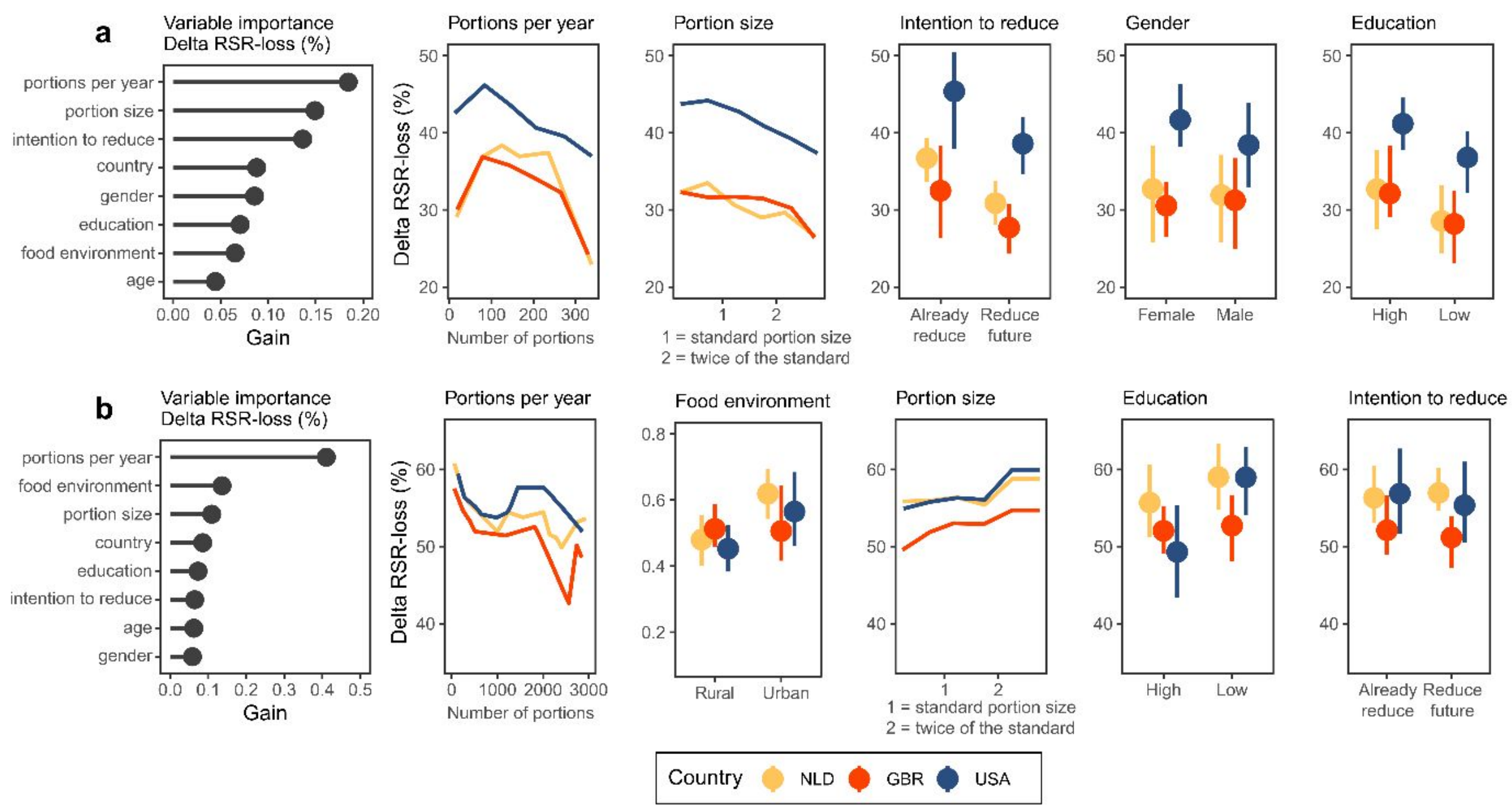

Fig. S9 | Prediction of delta RSR-loss (%) for respondents who intend to reduce their meat or dairy intake. **a**, Meat and meat alternatives. The leftmost panel depicts the importance of the variables included in the model, where gain describes the increase in predictive power if the variable is included in the model. The following five panels show the response plots for the five most important variables grouped by country **b**, Dairy and dairy alternatives. The response plots are ordered by variable importance for meat and meat alternatives (**a**) and dairy and dairy alternatives (**b**). The error bars represent the 90% percentile interval derived from bootstrap resampling.
