# Supplementary material for: Potential Biodiversity Footprint Reductions from Intended Shifts to Plant-Based Meals
Source: Environ Sci Technol. 2026 Sep 24;60(39):27743–53. doi: 10.1021/acs.est.6c02675 (PMC13648232; doi:10.1021/acs.est.6c02675)
Supplement: Supplementary file 2 [file es6c02675_si_002.pdf]

UK version

All Questions

Screen1

Question 1 (Welcome)

Welcome to this online survey on eating habits and behaviours. Thank you for participating!

You have received this questionnaire because you successfully completed the screener and you meet the selection criteria of the study.

Filling in this questionnaire will take approx. 15 minutes.

We are interested in your personal opinion, therefore there are no right or wrong answers.

Did you read all the information above?

Please click "Next".

Question 2 (source) (Q1)

Question 3 (state) (Q2)

Screen2

Question 1 (1.0 Eating habit) (Q3)

Which of the following BEST describes your usual eating habits?

- ◯ I eat meat and fish (1)
- ◯ I do eat meat, but I don't eat fish (2)
- ◯ I do not eat meat, but I do eat fish (3)
- ◯ I do not eat any meat/fish, but I do eat dairy and eggs (4)
- ◯ I do not eat any meat/fish, dairy and eggs (5)

Screen3

Only ask this screen when:

1 . Not Equals I do not eat meat, but I do eat fish (3) and

2 . 1.0 Eating habit Not Equals Ik eet geen vis of vlees, maar ik eet wel zuivel en eieren (4) and

3 . 1.0 Eating habit Not Equals Ik eet geen vlees, vis en zuivel (5)

Question 1 (2.1_General_meat_consumption) (Q4)

Thinking of your habitual dietary intake during the last month, how often do you consume meat as part of your main meal?

With 'main meal' we mean the biggest meal of the day, consumed for lunch or dinner. Often, this is the warm meal of the day.

- ◯ 6-7 times per week (1)
- ◯ 4-5 times per week (2)
- ◯ 2-3 times per week (3)
- ◯ once per week (4)
- ◯ 2-3 times per month (5)
- ◯ once per month (6)
- ◯ never or less than once per month (7)

Screen4

Only ask this screen when:

1 . Not Equals Nooit of minder vaak dan eens per maand (7) and

2 . Not Equals I do not eat meat, but I do eat fish (3) and

3 . 1.0 Eating habit Not Equals Ik eet geen vis of vlees, maar ik eet wel zuivel (4) and

4 . 1.0 Eating habit Not Equals Ik eet geen vlees, vis en zuivel (5)

Question 1 (intro)

You have indicated that you eat on average $$answer_display:2.1_General_meat_consumption$$ meat as part of your main meal.

With 'main meal' we mean the biggest meal of the day, consumed for lunch or dinner. Often, this is the warm meal of the day.

Question 2 (2.2.1 Beef consumption) (Q5)

Thinking of your habitual dietary intake during the last month, how often do you usually consume beef as part of your main meal?

never or less than once per month (1)	once per month (2)	2-3 times per month (3)	once per week (4)	2-3 times per week (5)	4-5 times per week (6)	6-7 times per week (7)

Question 3 (2.2.2 Poultry consumption) (Q6)

Thinking of your habitual dietary intake during the last month, how often do you usually consume chicken as part of your main meal?

never or less than once per month (1)	once per month (2)	2-3 times per month (3)	once per week (4)	2-3 times per week (5)	4-5 times per week (6)	6-7 times per week (7)

Question 4 (2.2.3 Pork consumption) (Q7)

Thinking of your habitual dietary intake during the last month, how often do you usually consume pork as part of your main meal?

never or less than once per month (1)	once per month (2)	2-3 times per month (3)	once per week (4)	2-3 times per week (5)	4-5 times per week (6)	6-7 times per week (7)

Question 5 (2.2.4 Lamb consumption) (Q8)

Thinking of your habitual dietary intake during the last month, how often do you usually consume lamb as part of your main meal?

never or less than once per month (1)	once per month (2)	2-3 times per month (3)	once per week (4)	2-3 times per week (5)	4-5 times per week (6)	6-7 times per week (7)

Screen5

Only ask this screen when:

1 . Not Equals Nooit of minder vaak dan eens per maand (7) and

2 . Not Equals I do not eat meat, but I do eat fish (3) and

3 . 1.0 Eating habit Not Equals Ik eet geen vis of vlees, maar ik eet wel zuivel (4) and

4 . 1.0 Eating habit Not Equals Ik eet geen vlees, vis en zuivel (5)

Question 1 (2.3 Portion size meat) (Q9)

From the pictures below, please select the portion size that best represents your habitual portion size of meat consumed in your main meal:

With 'main meal' we mean the biggest meal of the day, consumed for lunch or dinner. Often, this is the warm meal of the day.

○ 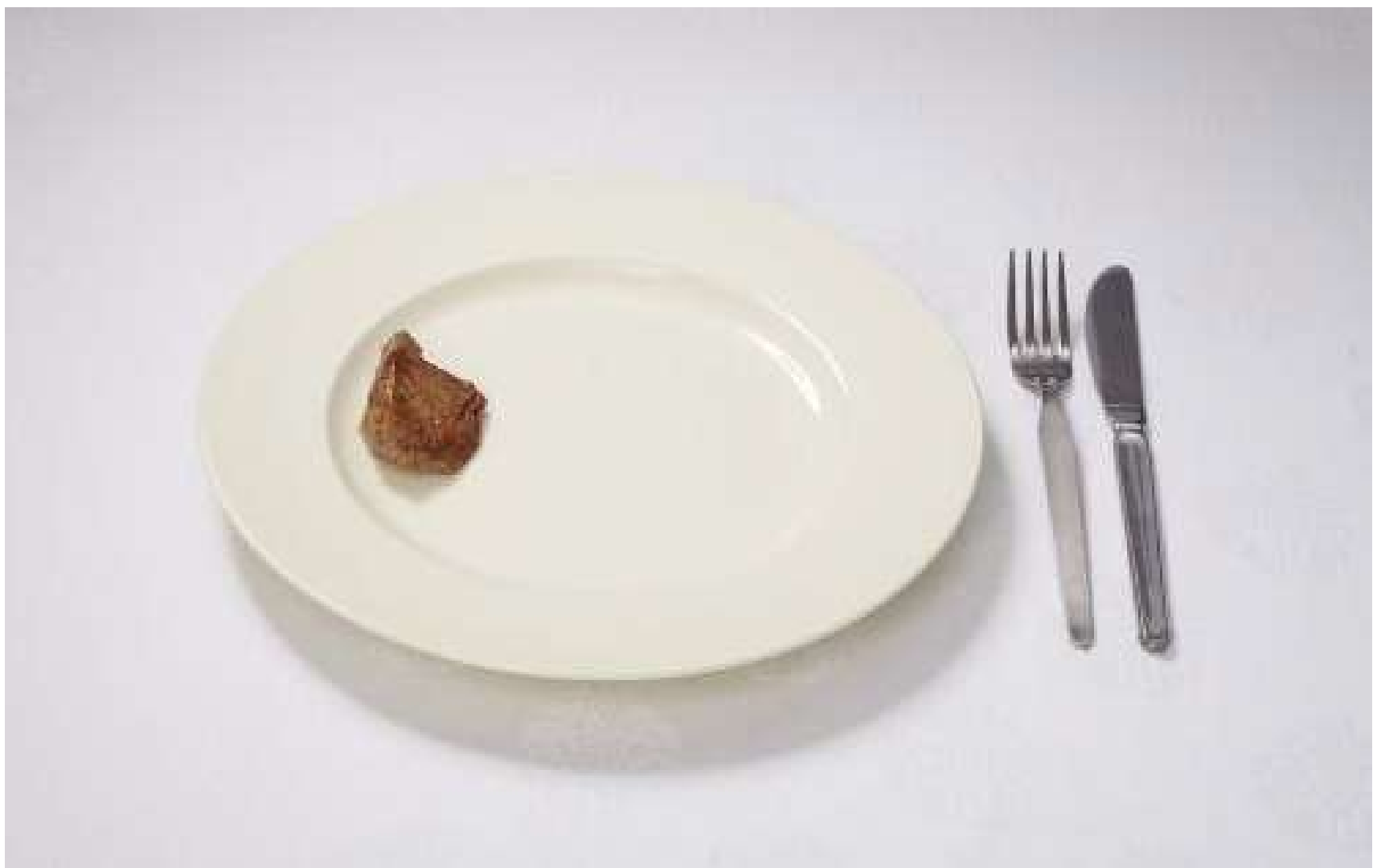

(1)

○ 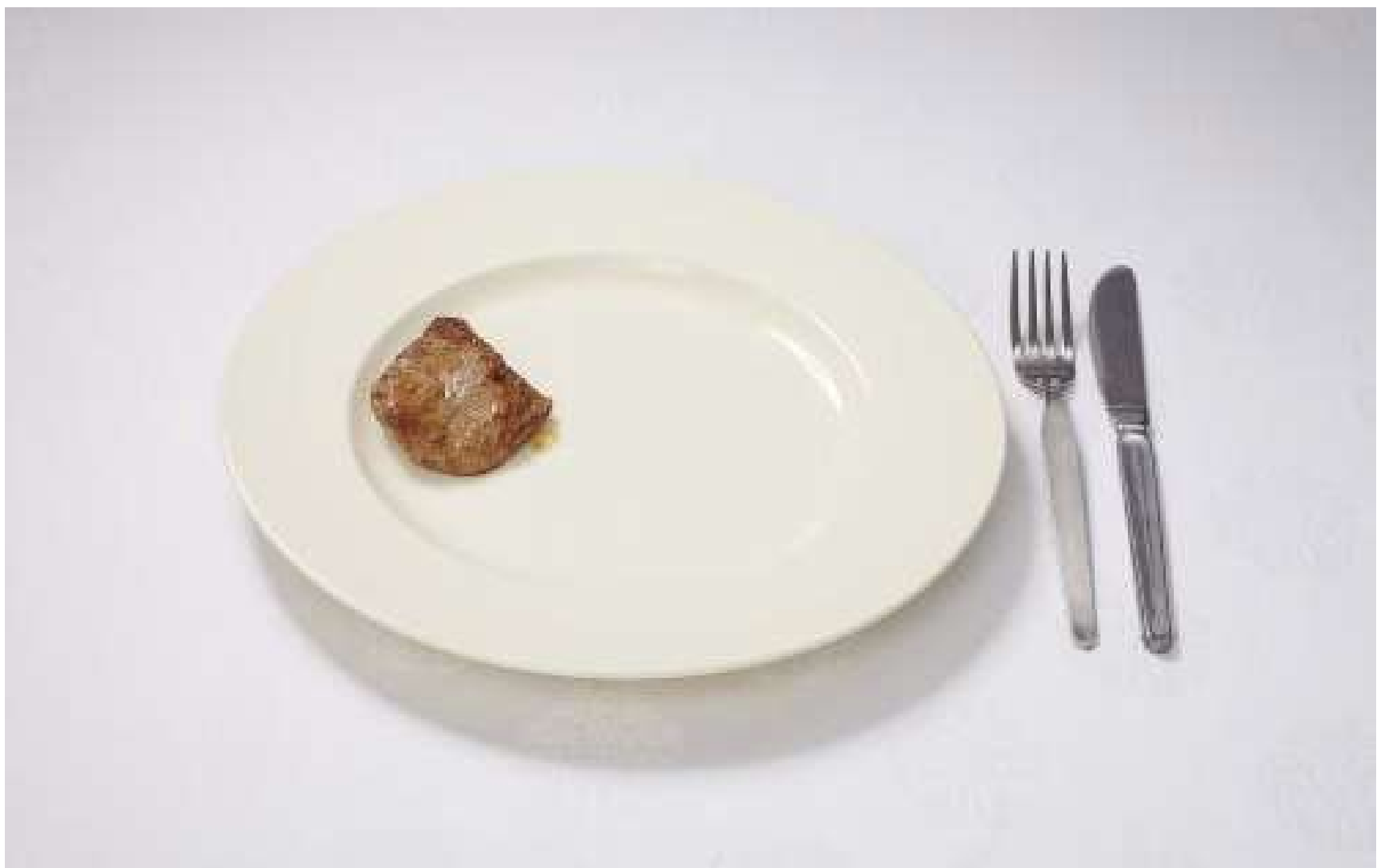

(2)

○ 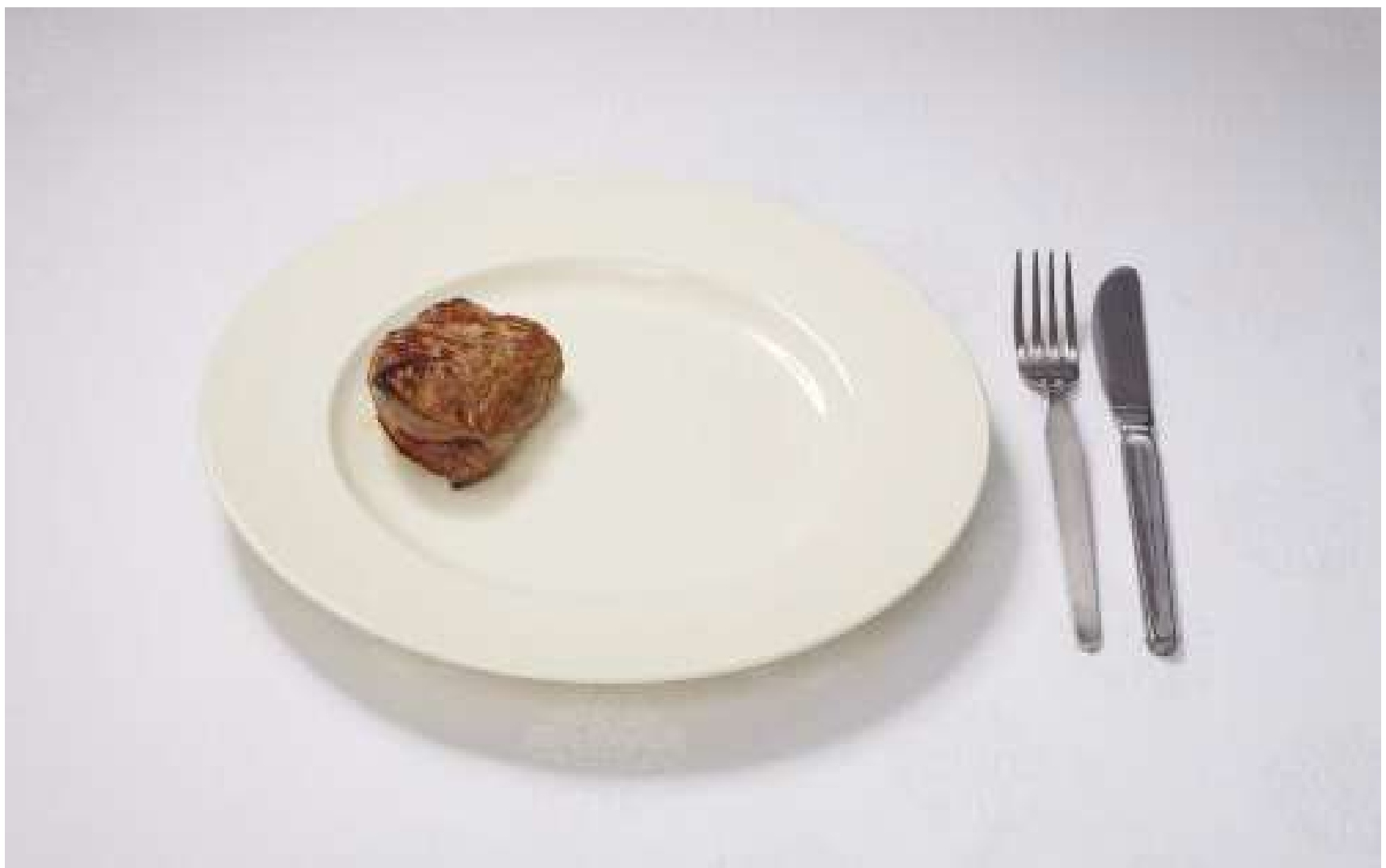

(3)

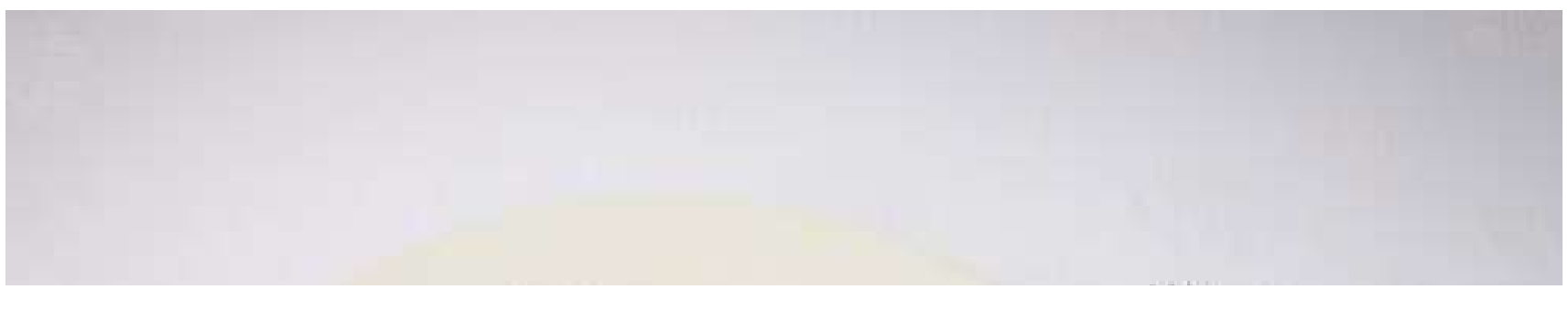

○ 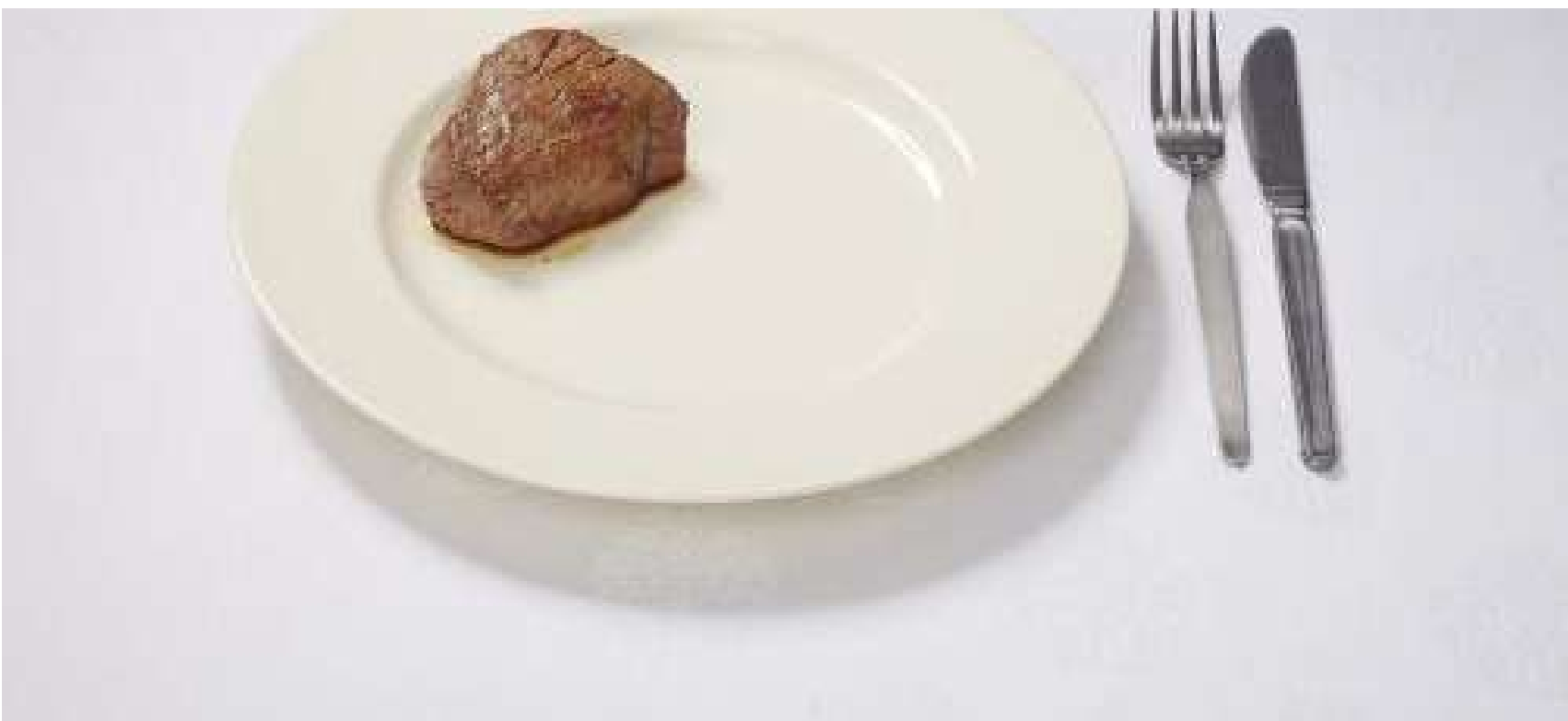

(4)

○ 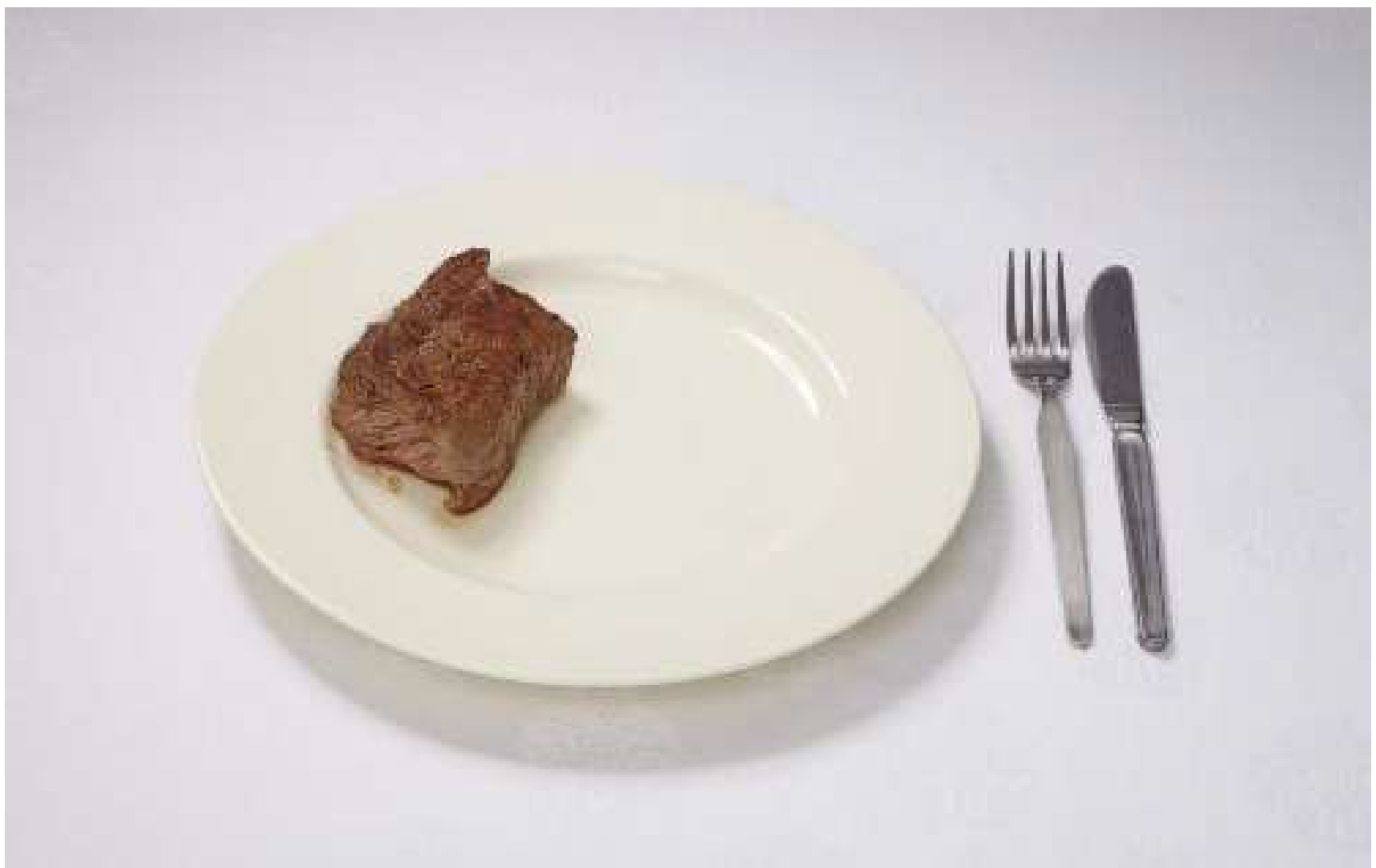

(5)

○ 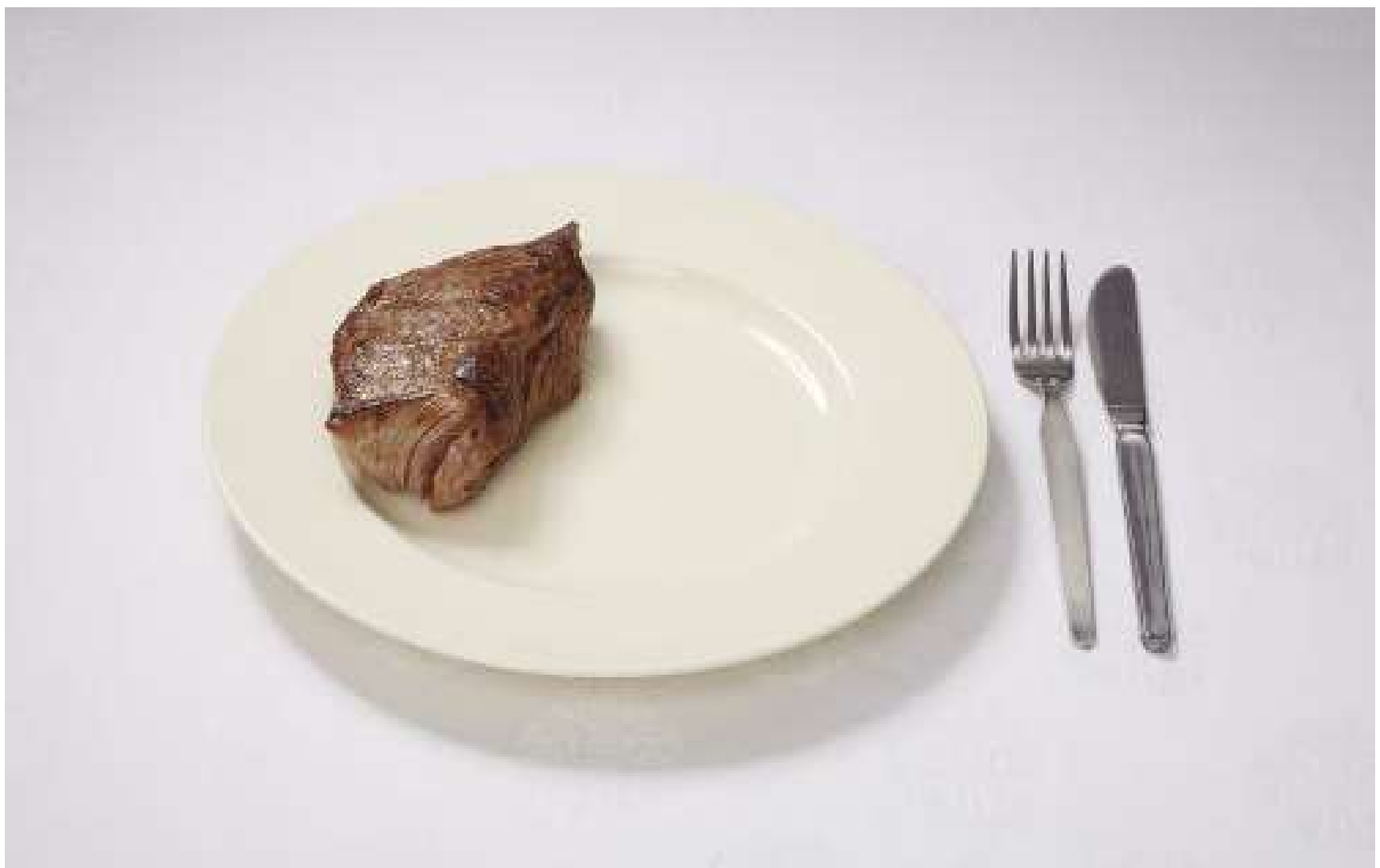

(6)

Screen6

Only ask this screen when:

1 . Not Equals I do not eat meat, but I do eat fish (3) and

2 . 1.0 Eating habit Not Equals Ik eet geen vis of vlees, maar ik eet wel zuivel (4) and

3 . 1.0 Eating habit Not Equals Ik eet geen vlees, vis en zuivel (5)

Question 1 (2.4 What foods for meat replacement) (Q10)

On the days that you do not consume meat with your main meal, do you deliberately replace it with the following foods?

Check all that apply. Multiple answers possible.

With 'main meal' we mean the biggest meal of the day, consumed for lunch or dinner. Often, this is the warm meal of the day.

Items randomized

- ☐ Fish or seafood (Fish_Seafood)Switch: **Fish_Seafood1**
- ☐ Eggs (Eggs)Switch: **Eggs1**
- ☐ Cheese or dairy items (Cheese_Dairy)Switch: **Cheese_Dairy1**
- ☐ Meat alternatives mimicking the taste & texture of meat (e.g. impossible burger of plant-based chicken) (Meat_alternatives)Switch: **Meat_alternatives1**
- ☐ Lentills, beans, or other pulses (incl. bean burgers, falafel etc.) (Legumes)Switch: **Legumes1**
- ☐ Tofu or tempeh (Tofu_Tempeh)Switch: **Tofu_Tempeh1**
- ☐ Nuts or seeds (e.g., cashew nuts, peanuts, pumpkin seeds) (Nuts_Seeds)Switch: **Nuts_Seeds1**
- ☐ Vegetables (e.g. mushrooms, broccoli, tomato) (Vegetables)Switch: **Vegetables1**
- ☐ Other, namely (other) Fixed!
- ☐ I do not deliberately replace meat with other foods in my main meal (none) Fixed!

Question 2 (2.5.1. Fish & Seafood Frequency replacement) (Q11)

You have indicated that you eat on average $$answer_display:2.1_General_meat_consumption$$ meat as part of your main meal.

Thinking of your habitual dietary intake in the past month, when consuming a main meal without meat, how often did you usually use <u>fish or seafood</u> to replace meat in your main meal?

Show on switch: **Fish_Seafood1**

never or less than once per month (1)	once per month (2)	2-3 times per month (3)	once per week (4)	2-3 times per week (5)	4-5 times per week (6)	6 times per week (7)

Question 3 (2.5.2. Eggs Frequency replacement) (Q12)

You have indicated that you eat on average $$answer_display:2.1_General_meat_consumption$$ meat as part of your main meal.

Thinking of your habitual dietary intake in the past month, when consuming a main meal without meat, how often did you usually use <u>eggs</u> to replace meat in your main meal?

Show on switch: **Eggs1**

never or less than once per month (1)	once per month (2)	2-3 times per month (3)	once per week (4)	2-3 times per week (5)	4-5 times per week (6)	6 times per week (7)

Question 4 (2.5.3. Cheese or dairy items Frequency replacement) (Q13)

Question 5 (2.5.4. Meat alternatives Frequency replacement) (Q14)

Question 6 (2.5.15. Legumes Frequency replacement) (Q15)

Question 7 (2.5.6. Tofu & Tempeh Frequency replacement) (Q16)

Question 8 (2.5.7. Nuts & Seeds Frequency replacement) (Q17)

Question 9 (2.5.8. Vegetables Frequency replacement) (Q18)

You have indicated that you eat on average $$answer_display:2.1_General_meat_consumption$$ meat as part of your main meal.

Thinking of your habitual dietary intake in the past month, when consuming a main meal without meat, how often did you usually use cheese or dairy items to replace meat in your main meal?

Show on switch: Cheese_Dairy1

never or less than once per month (1)	once per month (2)	2-3 times per month (3)	once per week (4)	2-3 times per week (5)	4-5 times per week (6)	6 times per week (7)

You have indicated that you eat on average $$answer_display:2.1_General_meat_consumption$$ meat as part of your main meal.

Thinking of your habitual dietary intake in the past month, when consuming a main meal without meat, how often did you usually use meat alternatives to replace meat in your main meal?

Show on switch: Meat_alternatives1

never or less than once per month (1)	once per month (2)	2-3 times per month (3)	once per week (4)	2-3 times per week (5)	4-5 times per week (6)	6 times per week (7)

You have indicated that you eat on average $$answer_display:2.1_General_meat_consumption$$ meat as part of your main meal.

Thinking of your habitual dietary intake in the past month, when consuming a main meal without meat, how often did you usually use lentils, bens or other pulses to replace meat in your main meal?

Show on switch: Legumes1

never or less than once per month (1)	once per month (2)	2-3 times per month (3)	once per week (4)	2-3 times per week (5)	4-5 times per week (6)	6 times per week (7)

You have indicated that you eat on average $$answer_display:2.1_General_meat_consumption$$ meat as part of your main meal.

Thinking of your habitual dietary intake in the past month, when consuming a main meal without meat, how often did you usually use tofu or tempeh to replace meat in your main meal?

Show on switch: Tofu_Tempeh1

never or less than once per month (7)	once per month (1)	2-3 times per month (2)	once per week (3)	2-3 times per week (4)	4-5 times per week (5)	6 times per week (6)

You have indicated that you eat on average $$answer_display:2.1_General_meat_consumption$$ meat as part of your main meal.

Thinking of your habitual dietary intake in the past month, when consuming a main meal without meat, how often did you usually use to nuts or seeds to replace meat in your main meal?

Show on switch: Nuts_Seeds1

never or less than once per month (1)	once per month (2)	2-3 times per month (3)	once per week (4)	2-3 times per week (5)	4-5 times per week (6)	6 times per week (7)

You have indicated that you eat on average $$answer_display:2.1_General_meat_consumption$$ meat as part of your main meal.

Thinking of your habitual dietary intake in the past month, when consuming a main meal without meat, how often did you usually use <u>vegetables</u> to replace meat in your main meal?

Show on switch: **Vegetables1**

never or less than once per month (1)	once per month (2)	2-3 times per month (3)	once per week (4)	2-3 times per week (5)	4-5 times per week (6)	6 times per week (7)

Screen7

Only ask this screen when:

1 . 1.0 Eating habit Not Equals Ik eet geen vlees, vis en zuivel (5) and

2 . 1.0 Eating habit Not Equals Ik eet geen vis of vlees, maar ik eet wel zuivel (4) and

3 . Not Equals I do not eat meat, but I do eat fish (3)

Question 1 (3.1 Attitude towards meat consumption) (Q19)

Which of the following statements <u>BEST</u> describes your approach towards meat consumption?

- ◯ I am not interested in lowering my meat intake and I have no intention of doing that within the next six months. (1)
- ◯ I am interested in lowering my meat intake and I have the intention of doing that within the next six months. (2)
- ◯ I am interested in lowering my meat intake and I have the intention of doing that within the next month. (3)
- ◯ I am interested in lowering my meat intake and I have started lowering my met intake during the last six months. (4)
- ◯ I am interested in lowering my meat intake and I have already lowered my meat intake for longer than six months. (5)

Screen8

Only ask this screen when:

1 . 3.1 Attitude towards meat consumption Equals I am interested in lowering my meat intake and I have the intention of doing that within the next six months. (2) or

2 . 3.1 Attitude towards meat consumption Equals I am interested in lowering my meat intake and I have the intention of doing that within the next month. (3) or

3 . 3.1 Attitude towards meat consumption Equals I am interested in lowering my meat intake and I have started lowering my meat intake during the last six months. (4) or

4 . 3.1 Attitude towards meat consumption Equals I am interested in lowering my meat intake and I have already lowered my meat intake for longer than six months. (5)

Question 1 (4.1 Intentions in reducing meat) (Q20)

Question 2 (4.2.1 Intention reduce beef) (Q21)

Question 3 (4.2.2 Intention reduce chicken) (Q22)

Question 4 (4.2.3 Intention reduce pork) (Q23)

Question 5 (4.2.4 Intention reduce lamb) (Q24)

When thinking of (further) reducing your meat intake, how frequently do you intend to have a main meal without meat?

With 'main meal' we mean the biggest meal of the day, consumed for lunch or dinner. Often, this is the warm meal of the day.

You have indicated that you eat now on average $$answer_display:2.1_General_meat_consumption$$ meat as part of your main meal.

- ◯ I intend to completely stop eating meat during the main meal. (1)
- ◯ less often than once per month (2)
- ◯ once per month (3)
- ◯ 2-3 times per month (4)
- ◯ once per week (5)
- ◯ 2-3 times per week (6)
- ◯ 4-5 times per week (7)
- ◯ 6 times per week (8)

Please rate your intentions to reduce your intake of beef on the scale below:
(5-point scale with anchors: "1-do not intend to reduce at all" and "5-intend to reduce very much")

1 Do not intend to reduce at all (1)	2 (2)	3 (3)	4 (4)	5 Intend to reduce very much (5)

Please rate your intentions to reduce your intake of chicken on the scale below:
(5-point scale with anchors: "1-do not intend to reduce at all" and "5-intend to reduce very much")

1 Do not intend to reduce at all (1)	2 (2)	3 (3)	4 (4)	5 Intend to reduce very much (5)

Please rate your intentions to reduce your intake of pork on the scale below:
(5-point scale with anchors: "1-do not intend to reduce at all" and "5-intend to reduce very much")

1 Do not intend to reduce at all (1)	2 (2)	3 (3)	4 (4)	5 Intend to reduce very much (5)

Please rate your intentions to reduce your intake of lamb on the scale below:
(5-point scale with anchors: "1-do not intend to reduce at all" and "5-intend to reduce very much")

1 Do not intend to reduce at all (1)	2 (2)	3 (3)	4 (4)	5 Intend to reduce very much (5)

Question 6 (4.3 Intentions consumption meat alternative foods) (Q25)

Please rate your intentions to use the following foods to replace meat in your meal on the scale below:
(5-point scale with anchors: "1-do not intend to use at all" and "5-intend to use very much")
Objects randomized

	**1 Do not intend to use at all**	**2**	**3**	**4**	**5 Intend to use very much**
	(1)	(2)	(3)	(4)	(5)
Fish or seafood **(Fish_Seafood)(Q26)**	○	○	○	○	○
Eggs **(Eggs)(Q27)**	○	○	○	○	○
Cheese or dairy items **(Cheese_Dairy)(Q28)**	○	○	○	○	○
Meat alternatives mimicking the taste & texture of meat (e.g. impossible burger of plant-based chicken) **(Meat_alternatives)(Q29)**	○	○	○	○	○
Lentills, beans, or other pulses (incl. bean burgers, falafel etc.) **(Legumes)(Q30)**	○	○	○	○	○
Tofu or tempeh **(Tofu_Tempeh)(Q31)**	○	○	○	○	○
Nuts or seeds (e.g., cashew nuts, peanuts, pumpkin seeds) **(Nuts_Seeds)(Q32)**	○	○	○	○	○
Vegetables (e.g. mushrooms, broccoli, tomato) **(Vegetables)(Q33)**	○	○	○	○	○

Screen9

Only ask this screen when:

1 . Not Equals I do not eat meat, but I do eat fish (3) and

2 . 1.0 Eating habit Not Equals Ik eet geen vis of vlees, maar ik eet wel zuivel (4) and

3 . 1.0 Eating habit Not Equals Ik eet geen vlees, vis en zuivel (5)

Question 1 (5.2 Perceived Barriers Meat Reduction) (Q34)

Please indicate on the scale below to what extent you agree/disagree with the following statements:
(5-point scale with anchors: "1- completely disagree" and "5- completely agree")
Objects randomized

	1 Completely disagree	2	3	4	5 Completely agree
	(1)	(2)	(3)	(4)	(5)
I really enjoy the taste of meat **(Taste_Meat)(Q35)**	○	○	○	○	○
I really enjoy the texture of meat **(Texture_Meat)(Q36)**	○	○	○	○	○
I find that meals without meat are less filling **(Satiation_Meat)(Q37)**	○	○	○	○	○
I find plant-based meat alternatives (e.g., a vegetarian burger, falafel, tofu) less value for money **(Expensive_Meat)(Q38)**	○	○	○	○	○
I can easily find ingredients for meatless meals in the places where I usually buy food **(Accessibility_meatreplacers_Meat)(Q39)**	○	○	○	○	○
Eating meat is a habit for me **(Habit_Meat)(Q40)**	○	○	○	○	○
I like to try new, unfamiliar foods or dishes. **(Open_new_food_Meat)(Q41)**	○	○	○	○	○
People close to me (e.g. friends/family) support me in lowering my meat intake **(Peer_influence_Meat)(Q42)**	○	○	○	○	○
I know a lot of people who reduce their meat intake **(Relatives_reducing_meat_Meat)(Q43)**	○	○	○	○	○
I find it difficult to prepare meatless meals **(Culinary_Meat)**	○	○	○	○	○

(Q44)

I know a lot of meatless recipes **(Knowledge_meatless_recipes_Meat)(Q45)**	◯	◯	◯	◯	◯
I am afraid that I will miss out on important nutrients if I eat less meat **(Nutritional_Meat)(Q46)**	◯	◯	◯	◯	◯
I find meat alternatives, which mimic, the taste and texture of meat, unhealthy **(Unhealthy_Meatreplacers)(Q47)**	◯	◯	◯	◯	◯
Reducing meat intake is healthy **(Less_meat_healthy_Meat)(Q48)**	◯	◯	◯	◯	◯
Reducing meat intake is good for the planet **(Less_meat_sustainable_Meat)(Q49)**	◯	◯	◯	◯	◯
Eating meat and thinking about how it is produced evokes conflicting feelings in me **(Conflicting_feelings_Meat)(Q50)**	◯	◯	◯	◯	◯

Screen10

Only ask this screen when:

1 . Not Equals I do not eat meat, but I do eat fish (3) and

2 . 1.0 Eating habit Not Equals Ik eet geen vis of vlees, maar ik eet wel zuivel (4) and

3 . 1.0 Eating habit Not Equals Ik eet geen vlees, vis en zuivel (5)

Question 1 (Intro willingness to pay burgers)

Imagine you are shopping in a grocery store and have decided to buy burgers.

Please indicate on the scales below (by sliding the marker to the left or to the right) for each of the burgers, what is the maximum price that you usually would be willing to pay?

Question 2 (6.1.1. Meat Burger) (Q51)

Question 3 (6.1.2. Plant-based Burger) (Q52)

Meat burger

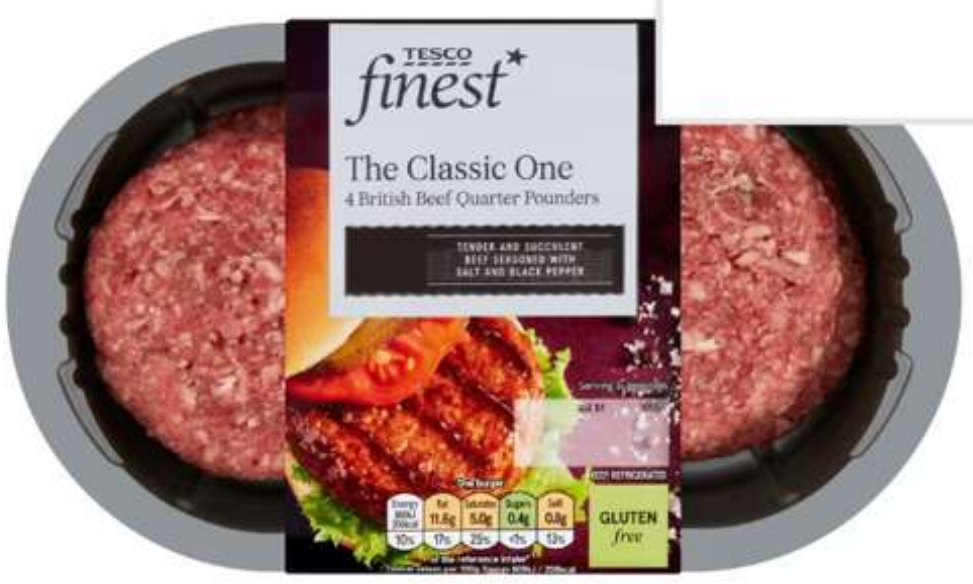

What is the maximum price that you would be willing to pay for four meat burgers?

£0,00 £4,00 £8,00

0.01

Plant-based burger that mimicks the taste and texture of meat

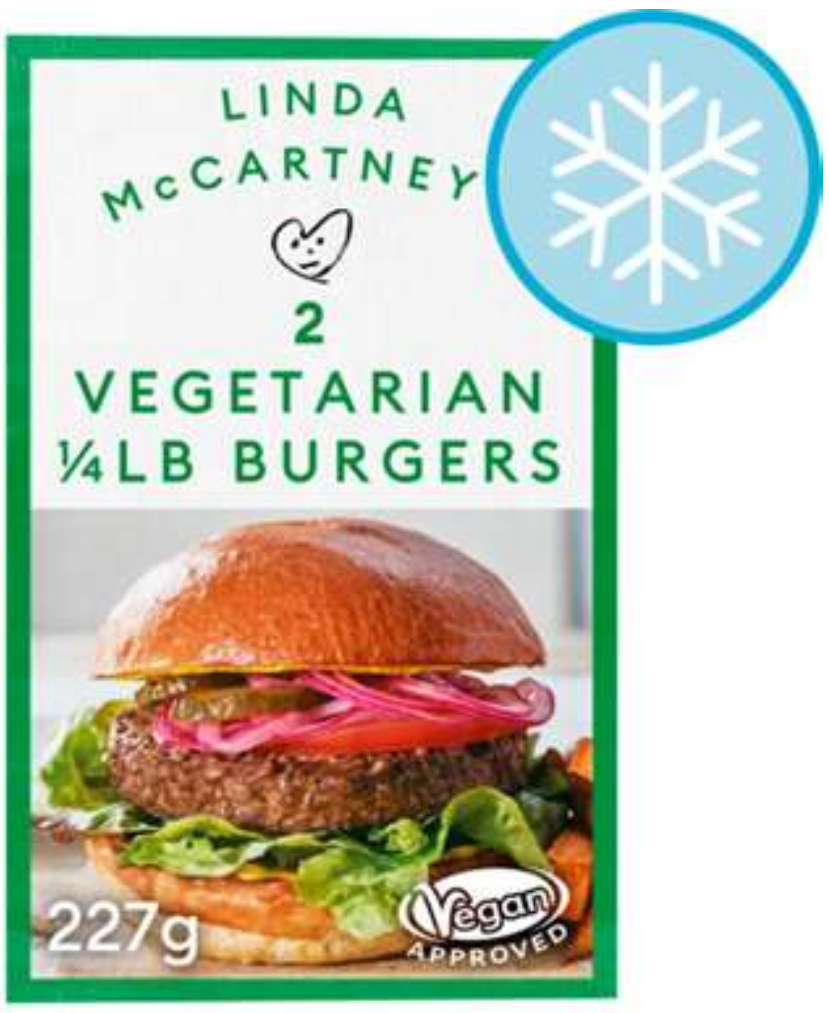

What is the maximum price that you would be willing to pay for four plant-based burgers that mimicks the taste and texture of meat?

£0,00 £4,00 £8,00

0.01

Question 4 (6.1.3. Bean Burger) (Q53)

Question 5 (6.1.4. Fish Burger) (Q54)

Question 6 (6.1.5. Cheese patties) (Q55)

Question 7 (6.1.6. Nuts & Seeds Burger) (Q56)

Question 8 (6.1.7. Vegetable burger) (Q57)

Lentil or bean burger

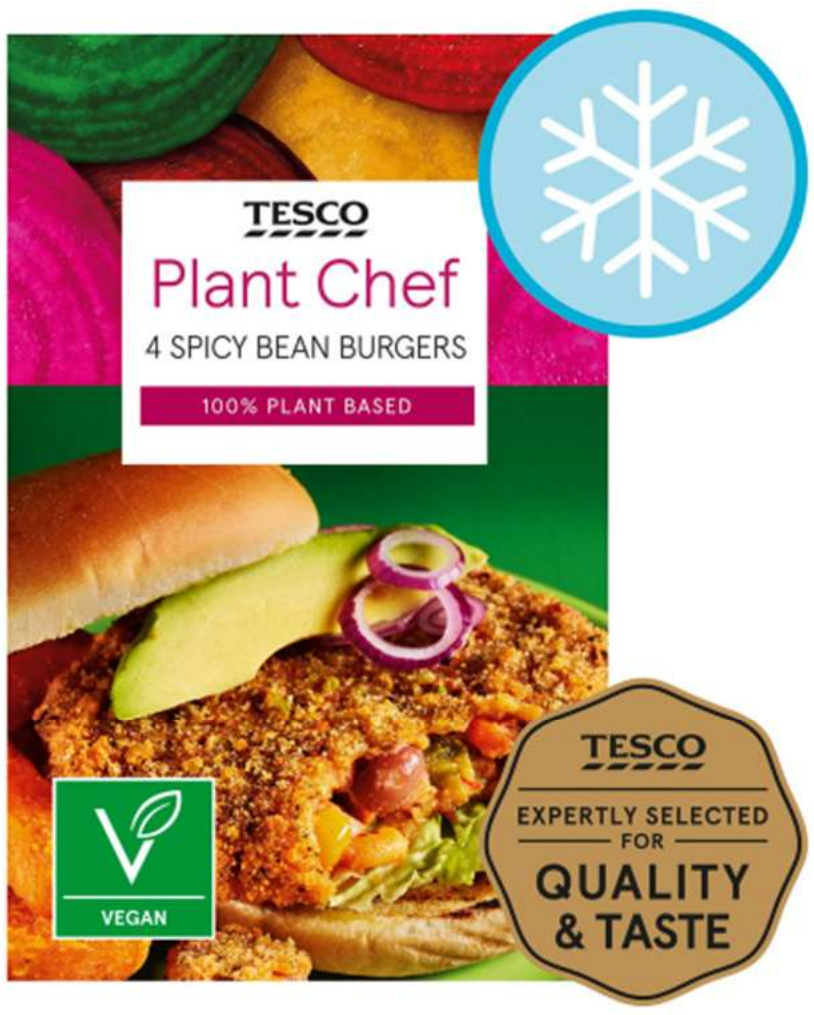

What is the maximum price that you would be willing to pay for <u>four lentil or bean burgers</u>?

£0,00 £4,00 £8,00

X 0.01

Fish burger

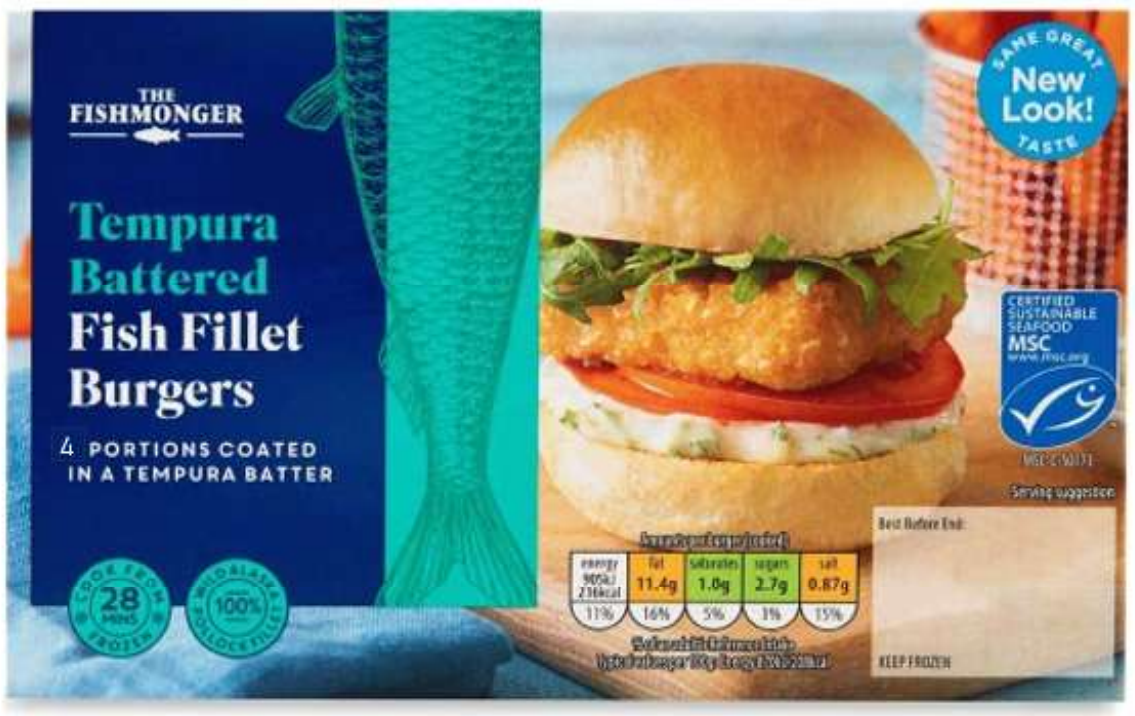

What is the maximum price that you would be willing to pay for <u>four fish burgers</u>?

£0,00 £4,00 £8,00

X 0.01

Burger made of cheese

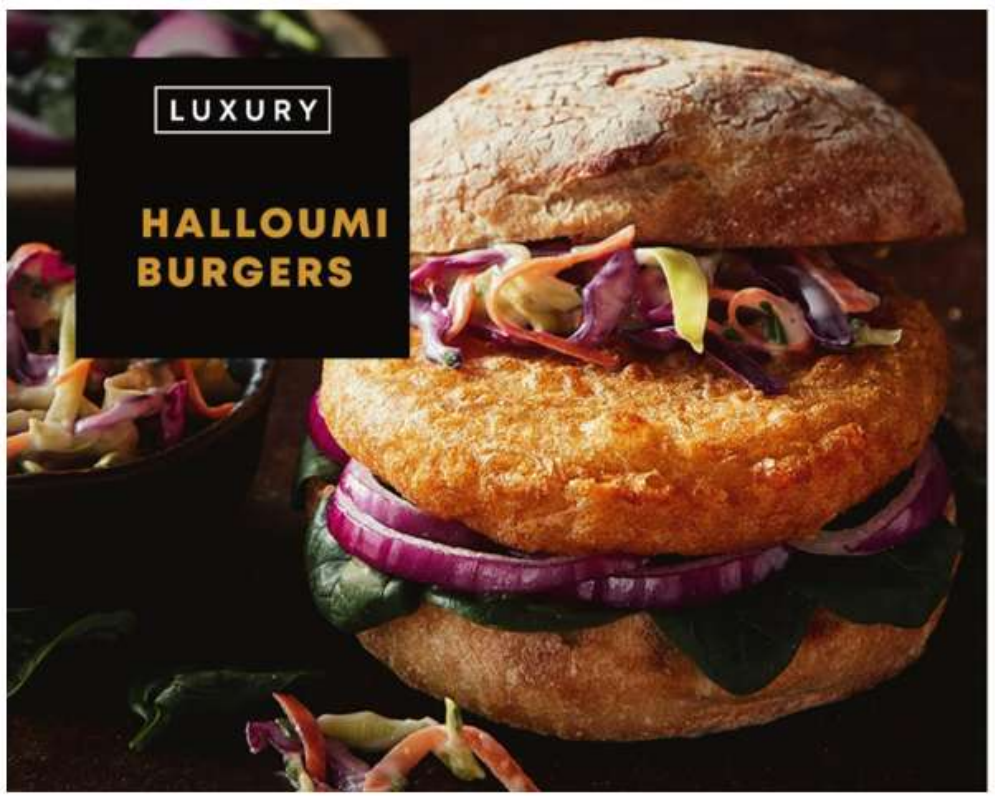

What is the maximum price that you would be willing to pay for **four burgers made of cheese**?

£0,00 £4,00 £8,00

0.01

Burgers made of nuts/seeds

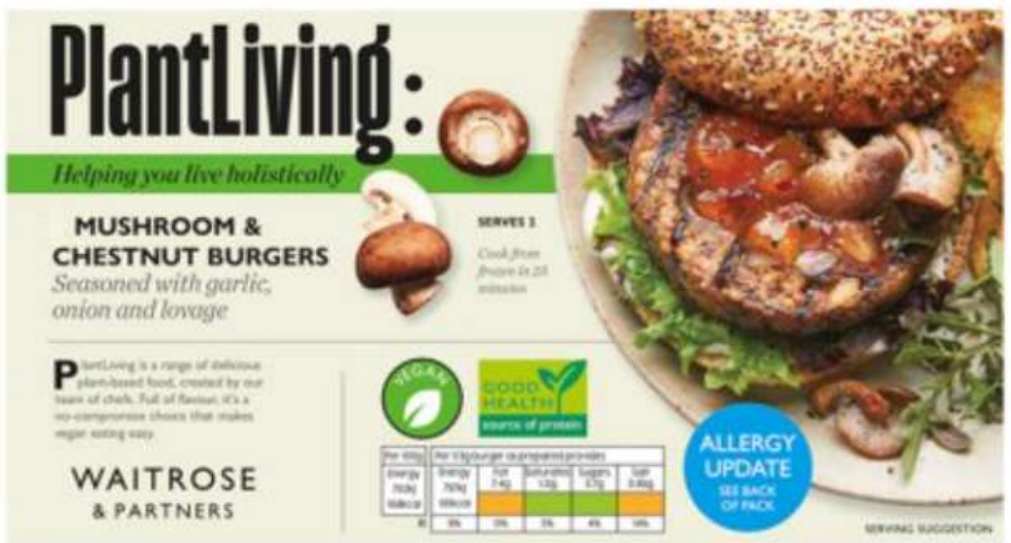

What is the maximum price that you would be willing to pay for **four burgers made of nuts/seeds**?

£0,00 £4,00 £8,00

0.01

Vegetable burger

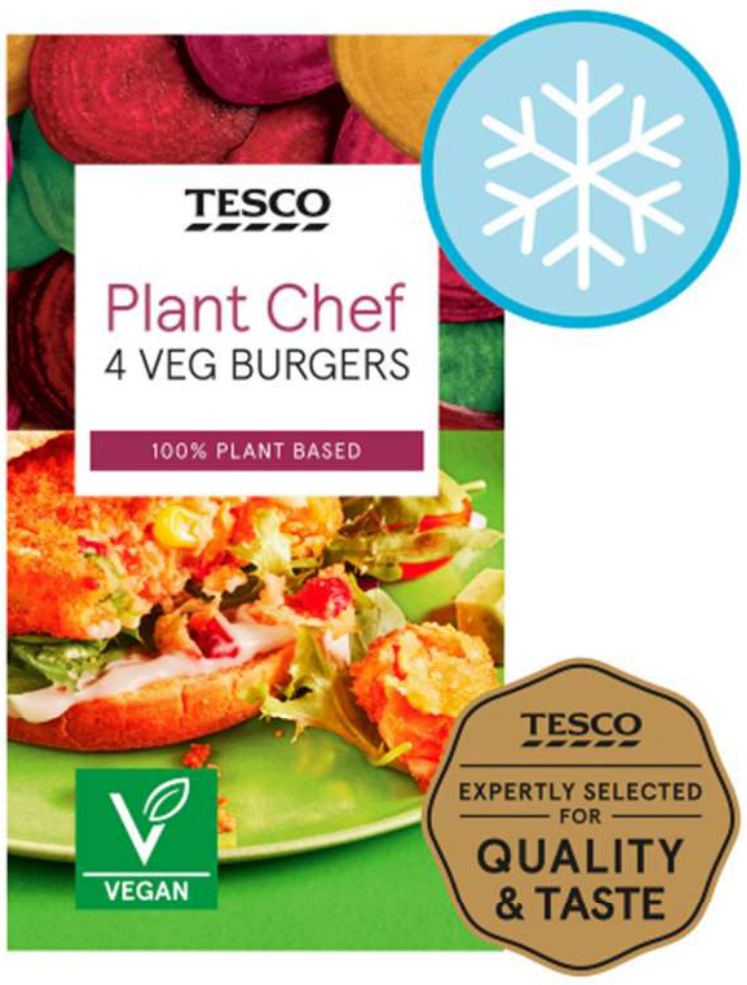

What is the maximum price that you would be willing to pay for **four vegetable burgers**?

£0,00 £4,00 £8,00

0.01

Screen11

Only ask this screen when:

1 . 1.0 Eating habit Not Equals Ik eet geen vlees, vis en zuivel (5)

Question 1 (7.0 intro)

Thinking of your habitual dietary intake during the last month, how often do you usually consume the dairy products below?

Question 2 (7.1.1 Consumption dairy drinks) (Q58)

Milk and other dairy drinks (e.g. buttermilk, yoghurt drink, kefir)

never or less than once per month (1)	1-3 times per month (2)	1-3 times per week (3)	4-6 times per week (4)	once per day (5)	2 times per day (6)	3 or more times per day (7)

Question 3 (7.1.2 Consumption yoghurt) (Q59)

Yoghurt or quark

never or less than once per month (1)	1-3 times per month (2)	1-3 times per week (3)	4-6 times per week (4)	once per day (5)	2 times per day (6)	3 or more times per day (7)

Question 4 (7.1.3 Consumption ice cream) (Q60)

Ice cream

never or less than once per month (1)	1-3 times per month (2)	1-3 times per week (3)	4-6 times per week (4)	once per day (5)	2 times per day (6)	3 or more times per day (7)

Question 5 (7.1.4 Consumption dairy desserts) (Q61)

Dairy desserts

never or less than once per month (1)	1-3 times per month (2)	1-3 times per week (3)	4-6 times per week (4)	once per day (5)	2 times per day (6)	3 or more times per day (7)

Question 6 (7.1.5 Consumption cheese) (Q62)

Cheese

never or less than once per month (1)	1-3 times per month (2)	1-3 times per week (3)	4-6 times per week (4)	once per day (5)	2 times per day (6)	3 or more times per day (7)

Screen12

Only ask this screen when:

1 . 1.0 Eating habit Not Equals Ik eet geen vlees, vis en zuivel (5)

Question 1 (7.2 What foods for dairy replacement) (Q63)

Do you usually use one or more of the following plant-based alternatives to deliberately replace dairy products?
With plant-based alternatives we mean products that are designed to be used in the same way as dairy products, e.g. soy milk instead of cow's milk or a vegan version of ice cream.

Select all that apply. Multiple answers possible.

- ☐ Plant-based milk (e.g. soy milk, almond milk, oat milk) (Dairy_drinks_Plantbased)Switch: **PBMilk1**
- ☐ Plant-based yoghurt or quark (Yoghurt_Quark_Plantbased)Switch: **PBYoghurt_Quark1**
- ☐ Plant-based ice cream (Ice_cream_Plantbased)Switch: **PBIcecream1**
- ☐ Plant-based alternatives for dairy desserts (Dairy_dessert_Plantbased)Switch: **PBDairyDesserts1**
- ☐ Plant-based cheese (Cheese_Plantbased)Switch: **PBCheese1**
- ☐ Other, namely (other) Fixed!
- ☐ No, I do not use plant-based alternatives to replace dairy products (none) Fixed!

Question 2 (7.3.1. Milk Plant-based Frequency replacement) (Q64)

You have indicated that you consume on average $$answer_display:7.1.1 Consumption dairy drinks$$ dairy drinks.

Thinking of your habitual dietary intake during the last month, how often did you usually use <u>plant-based milk</u> to replace dairy products?
Show on switch: **PBMilk1**

never or less than once per month (1)	1-3 times per month (2)	1-3 times per week (3)	4-6 times per week (4)	once per day (5)	2 times per day (6)	3 or more times per day (7)

Question 3 (7.3.2. Yoghurt & Quark Plant-based Frequency replacement) (Q65)

You have indicated that you consume on average $$answer_display:7.1.2 Consumption yoghurt$$ yoghurt or quark.

Thinking of your habitual dietary intake during the last month, how often did you usually use <u>plant-based yoghurt or quark</u> to replace dairy products?
Show on switch: **PBYoghurt_Quark1**

never or less than once per month (1)	1-3 times per month (2)	1-3 times per week (3)	4-6 times per week (4)	once per day (5)	2 times per day (6)	3 or more times per day (7)

Question 4 (7.3.3. Ice cream Plant-based Frequency replacement) (Q66)

You have indicated that you consume on average $$answer_display:7.1.3 Consumption ice cream$$ ice cream.

Thinking of your habitual dietary intake during the last month, how often did you usually use <u>plant-based ice cream</u> to replace dairy products?
Show on switch: **PBIcecream1**

never or less than once per month (1)	1-3 times per month (2)	1-3 times per week (3)	4-6 times per week (4)	once per day (5)	2 times per day (6)	3 or more times per day (7)

Question 5 (7.3.4. Dairy Desserts Plant-based Frequency replacement) (Q67)

Question 6 (7.3.5. Cheese Plant-based Frequency replacement) (Q68)

You have indicated that you consume on average $$answer_display:7.1.4 Consumption dairy desserts$$ dairy desserts.

Thinking of your habitual dietary intake during the last month, how often did you usually use <u>plant-based dairy desserts</u> to replace dairy products?
Show on switch: **PBDairyDesserts1**

never or less than once per month (1)	1-3 times per month (2)	1-3 times per week (3)	4-6 times per week (4)	once per day (5)	2 times per day (6)	3 or more times per day (7)

You have indicated that you consume on average $$answer_display:7.1.5 Consumption cheese$$ cheese.

Thinking of your habitual dietary intake during the last month, how often did you usually use <u>plant-based cheese</u> to replace dairy products?
Show on switch: **PBCheese1**

never or less than once per month (1)	1-3 times per month (2)	1-3 times per week (3)	4-6 times per week (4)	once per day (5)	2 times per day (6)	3 or more times per day (7)

Screen13

Only ask this screen when:

1 . 1.0 Eating habit Not Equals Ik eet geen vlees, vis en zuivel (5)

Question 1 (8.1 Attitude towards dairy consumption) (Q69)

Which of the following statements <u>BEST</u> describes your approach towards consumption of dairy products?

- ◯ I am not interested in lowering my intake of dairy products and I have no intention of doing that within the next six months. (1)
- ◯ I am interested in lowering my intake of dairy products and I have the intention of doing that within the next six months. (2)
- ◯ I am interested in lowering my intake of dairy products and I have the intention of doing that within the next month. (3)
- ◯ I am interested in lowering my intake of dairy products and I have started lowering my intake of dairy products during the last six months. (4)
- ◯ I am interested in lowering my intake of dairy products and I have already lowered my intake of dairy products for longer than six months. (5)

Screen14

Only ask this screen when:

1 . Equals I am interested in lowering my intake of dairy products and I have the intention of doing that within the next six months. (2) or

2 . 8.1 Attitude towards dairy consumption Equals I am interested in lowering my intake of dairy products and I have the intention of doing that within the next month. (3) or

3 . 8.1 Attitude towards dairy consumption Equals I am interested in lowering my intake of dairy products and I have started lowering my intake of dairy products during the last six months. (4) or

4 . 8.1 Attitude towards dairy consumption Equals I am interested in lowering my intake of dairy products and I have already lowered my intake of dairy products for longer than six months. (5)

Question 1 (9.1.1. Intention reduce dairy drinks) (Q70)

Do you intend to reduce your intake of milk and other dairy drinks (e.g. buttermilk, yoghurt, kefir)?

yes (1)	no (2)

Question 2 (9.1.2 Intention reduce yoghurt quark) (Q71)

Do you intend to reduce your intake of yoghurt or quark?

yes (1)	no (2)

Question 3 (9.1.3 Intention reduce ice cream) (Q72)

Do you intend to reduce your intake of ice cream?

yes (1)	no (2)

Question 4 (9.1.4 Intention reduce dairy desserts) (Q73)

Do you intend to reduce your intake of dairy desserts?

yes (1)	no (2)

Question 5 (9.1.5 Intention reduce cheese) (Q74)

Do you intend to reduce your intake of cheese?

yes (1)	no (2)

Screen15

Only ask this screen when:

1 . 9.1.1. Intention reduce dairy drinks Equals yes (1) or

2 . 9.1.2 Intention reduce yoghurt quark Equals yes (1) or

3 . 9.1.3 Intention reduce ice cream Equals yes (1) or

4 . 9.1.4 Intention reduce dairy desserts Equals yes (1) or

5 . 9.1.5 Intention reduce cheese Equals yes (1)

Question 1 (9.2.1. Milk Frequency consumption) (Q75)

You have indicated that you consume on average $$answer_display:7.1.1 Consumption dairy drinks$$ dairy drinks and that you have the intention to reduce your intake of dairy drinks.

How frequently do you intend to still consume milk and other dairy drinks?

I intend to completely stop consuming dairy drinks (1)	once per month or less (2)	2-3 times per month (3)	1-3 times per week (4)	4-6 times per week (5)	once per day (6)	2 times per day (7)	3 or more times per day (8)

Question 2 (9.3.1. Milk Intention Frequency replacement) (Q76)

Please rate your intentions to use plant-based milk or other plant-based drinks to replace cow's milk and other dairy drinks:
(5-point scale with anchors: "1-do not intend to use at all" and "5-intend to use very much")

1 do not intend to use at all (1)	2 (2)	3 (3)	4 (4)	5 intend to use very much (5)

Question 3 (9.2.2. Yoghurt & Quark Frequency consumption) (Q77)

You have indicated that you consume on average $$answer_display:7.1.2 Consumption yoghurt$$ yoghurt or quark and that you have the intention to reduce your intake of dairy-based yoghurt or quark.

How frequently do you intend to still consume dairy-based yoghurt or quark?

I intend to completely stop consuming dairy-based yoghurt or quark (1)	once per month or less (2)	2-3 times per month (3)	1-3 times per week (4)	4-6 times per week (5)	once per day (6)	2 times per day (7)	3 or more times per day (8)

Question 4 (9.3.2. Yoghurt & Quark Intention Frequency replacement) (Q78)

Please rate your intentions to use plant-based yoghurt or quark to replace dairy-based yoghurt or quark:
(5-point scale with anchors: "1-do not intend to use at all" and "5-intend to use very much")

1 do not intend to use at all (1)	2 (2)	3 (3)	4 (4)	5 intend to use very much (5)

Question 5 (9.2.3. Ice cream Frequency consumption) (Q79)

You have indicated that you consume on average $$answer_display:7.1.3 Consumption ice cream$$ dairy-based ice cream and that you have the intention to reduce your intake of dairy-based ice cream.

How frequently do you intend to still consume dairy-based ice cream?

I intend to completely stop consuming dairy-based ice cream (1)	once per month or less (2)	2-3 times per month (3)	1-3 times per week (4)	4-6 times per week (5)	once per day (6)	2 times per day (7)	3 or more times per day (8)

Question 6 (9.3.2. Ice cream Intention Frequency replacement) (Q80)

Please rate your intentions to use plant-based ice cream to replace dairy-based ice cream:
(5-point scale with anchors: "1-do not intend to use at all" and "5-intend to use very much")

1 do not intend to use at all (1)	2 (2)	3 (3)	4 (4)	5 intend to use very much (5)

Question 7 (9.2.4. Dairy desserts Frequency consumption) (Q81)

Question 8 (9.3.2. Dairy Desserts Intention Frequency replacement) (Q82)

You have indicated that you consume on average $$answer_display:7.1.4 Consumption dairy desserts$$ dairy desserts and that you have the intention to reduce your intake of dairy desserts.

How frequently do you intend to still consume dairy desserts?

I intend to completely stop consuming dairy desserts (1)	once per month or less (2)	2-3 times per month (3)	1-3 times per week (4)	4-6 times per week (5)	once per day (6)	2 times per day (7)	3 or more times per day (8)

Please rate your intentions to use plant-based alternatives for dairy desserts to replace dairy desserts:
(5-point scale with anchors: "1-do not intend to use at all" and "5-intend to use very much")

1 do not intend to use at all (1)	2 (2)	3 (3)	4 (4)	5 intend to use very much (5)

Question 9 (9.2.5. Cheese Frequency consumption) (Q83)

You have indicated that you consume on average $$answer_display:7.1.5 Consumption cheese$$ dairy-based cheese and that you have the intention to reduce your intake of dairy-based cheese.

How frequently do you intend to still consume dairy-based cheese?

I intend to completely stop consuming dairy-based cheese (1)	once per month or less (2)	2-3 times per month (3)	1-3 times per week (4)	4-6 times per week (5)	once per day (6)	2 times per day (7)	3 or more times per day (8)

Question 10 (9.3.2. Cheese Intention Frequency replacement) (Q84)

Please rate your intentions to use plant-based cheese to replace dairy-based cheese?
(5-point scale with anchors: "1-do not intend to use at all" and "5-intend to use very much")

1 do not intend to use at all (1)	2 (2)	3 (3)	4 (4)	5 intend to use very much (5)

Screen16

Only ask this screen when:

1 . 1.0 Eating habit Not Equals Ik eet geen vlees, vis en zuivel (5)

Question 1 (10.2 Perceived Barriers Dairy Reduction) (Q85)

Please indicate on the scale below to what extent you agree/disagree with the following statements:
(5-point scale with anchors: "1-completely disagree" and "5- completely agree")
Objects randomized

	**1 Completely disagree**	**2**	**3**	**4**	**5 Completely agree**
	(1)	(2)	(3)	(4)	(5)
I really enjoy the taste of dairy products **(Taste_Dairy)(Q86)**	○	○	○	○	○
I really enjoy the texture of dairy products **(Texture_Dairy)(Q87)**	○	○	○	○	○
I find that plant-based dairy alternatives are less filling **(Satiation_Dairy)(Q88)**	○	○	○	○	○
I find plant-based dairy alternatives are less value for money **(Lessvaluable_Dairy)(Q89)**	○	○	○	○	○
I can easily find plant-based dairy alternatives in the places where I usually buy food **(Accessibility_Dairy)(Q90)**	○	○	○	○	○
Consuming dairy products is a habit for me **(Habit_Dairy)(Q91)**	○	○	○	○	○
Close others (e.g. family/friends) support me in lowering my intake of dairy products **(Peer_influence_Dairy)(Q92)**	○	○	○	○	○
I know a lot of people who reduce their intake of dairy products **(Relatives_reduce_dairy_Dairy)(Q93)**	○	○	○	○	○
I find it difficult to use plant-based dairy alternatives **(Culinary_Dairy)(Q94)**	○	○	○	○	○
I know a lot of applications where I	○	○	○	○	○

can use dairy alternatives **(Knowledge_Dairy)(Q95)**					
I am afraid that I will miss out on important nutrients if I consume fewer dairy products **(Nutritional_Dairy)(Q96)**	○	○	○	○	○
I like to try new, unfamiliar foods or dishes **(New_food_Dairy)(Q97)**	○	○	○	○	○
I find plant-based dairy alternatives unhealthy **(Processed_unhealthy_Dairy)(Q98)**	○	○	○	○	○
Reducing the intake of dairy products is healthy **(Less_dairy_healthy_Dairy)(Q99)**	○	○	○	○	○
Reducing the intake of dairy products is good for the planet **(Less_dairy_sustainable-Dairy)(Q100)**	○	○	○	○	○
Consuming dairy products and thinking about how they are produced evokes conflicting feelings in me **(Conflicting_feelings_Dairy)(Q101)**	○	○	○	○	○

Screen17 Randomgroup A

Only ask this screen when:

1 . 7.1.1 Consumption dairy drinks Greater than Nooit of minder vaak dan eens per maand (1)

Question 1 (intro)

Imagine you are shopping in a grocery store and have decided to buy milk.

Please indicate on the scales below (by sliding the marker to the left or to the right) for each of the products what is the maximum price that you usually would be willing to pay?

Question 2 (11.1.1. Cow's milk) (Q102)

Cow's milk

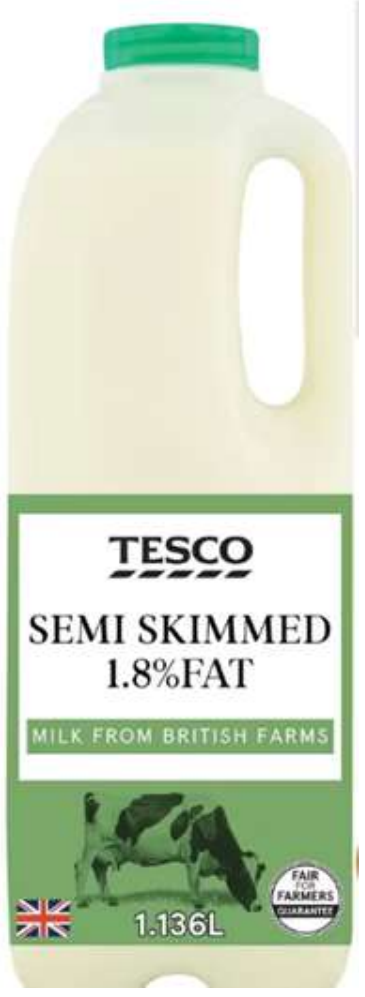

What is the maximum price that you would be willing to pay for 1,136 L/2 pints of milk?

£0,00 £1,20 £2,40

X **0.01**

Question 3 (11.1.2. Plant-based Milk) (Q103)

Plant-based milk

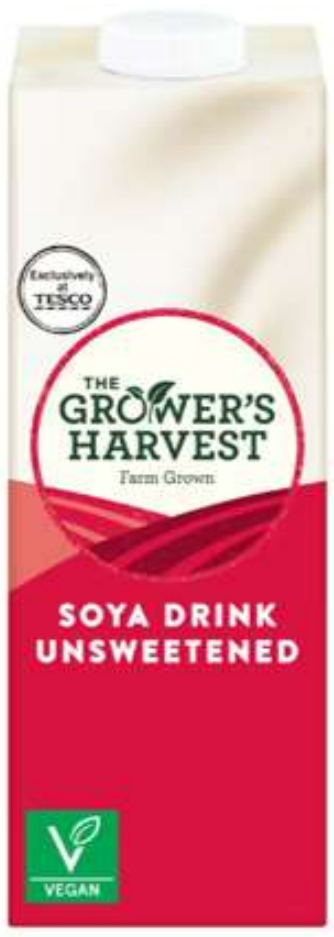

What is the maximum price that you would be willing to pay for 1,136 L/2 pints of plant-based milk?

£0,00 £1,20 £2,40

X **0.01**

Screen18 Randomgroup A

Only ask this screen when:

1 . 7.1.5 Consumption cheese Greater than Nooit of minder vaak dan eens per maand (1)

Question 1 (intro)

Imagine you are shopping in a grocery store and have decided to buy cheese.

Please indicate on the scales below (by sliding the marker to the left or to the right) for each of the products what is the maximum price that you usually would be willing to pay?

Question 2 (11.2.1. Cow's Cheese) (Q104)

Dairy cheese

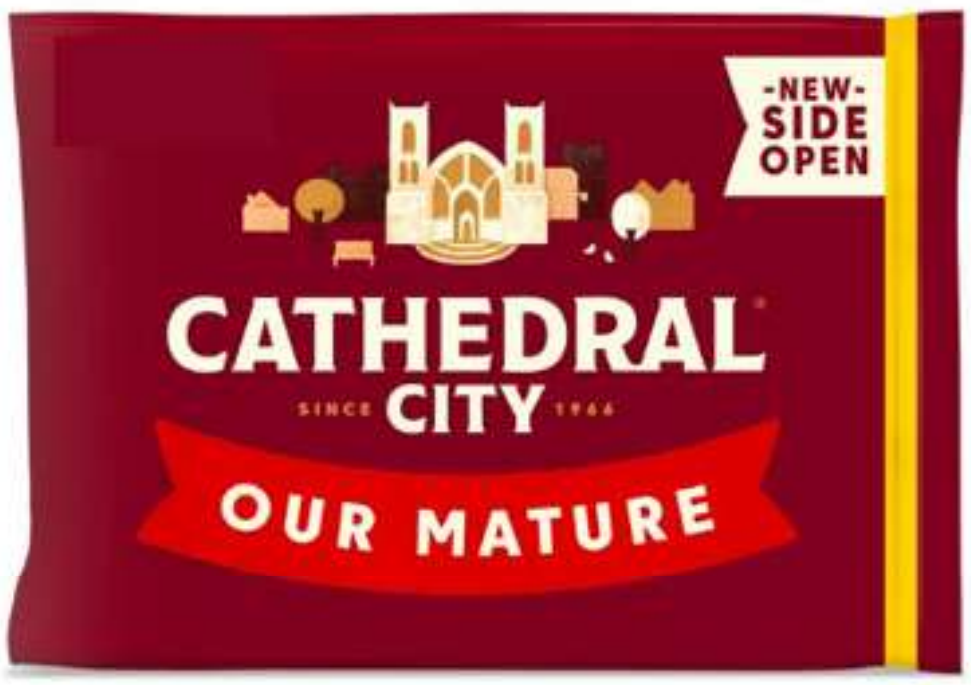

What is the maximum price that you would be willing to pay for 350 gram cheese?

£0,00 £4,75 £9,50

0.01

Question 3 (11.2.2. Plant-based Cheese) (Q105)

Plant-based cheese

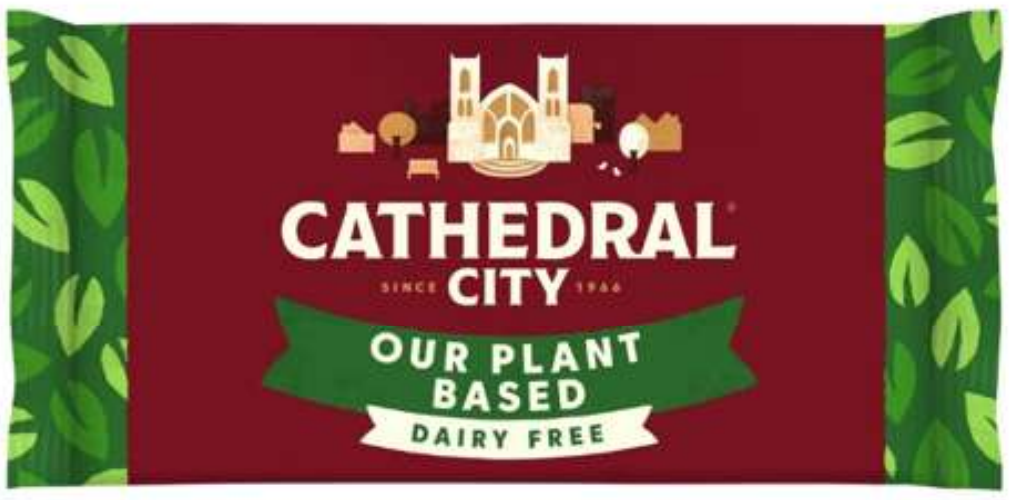

What is the maximum price that you would be willing to pay for 350 gram plant-based cheese?

£0,00 £4,75 £9,50

Screen19 Randomgroup A

Only ask this screen when:

1 . 7.1.3 Consumption ice cream Greater than Nooit of minder vaak dan eens per maand (1)

Question 1 (intro)

Imagine you are shopping in a grocery store and have decided to buy ice cream.

Please indicate on the scales below (by sliding the marker to the left or to the right) for each of the products what is the maximum price that you usually would be willing to pay?

Question 2 (11.3.1. Dairy Ice cream) (Q106)

Dairy-based ice cream

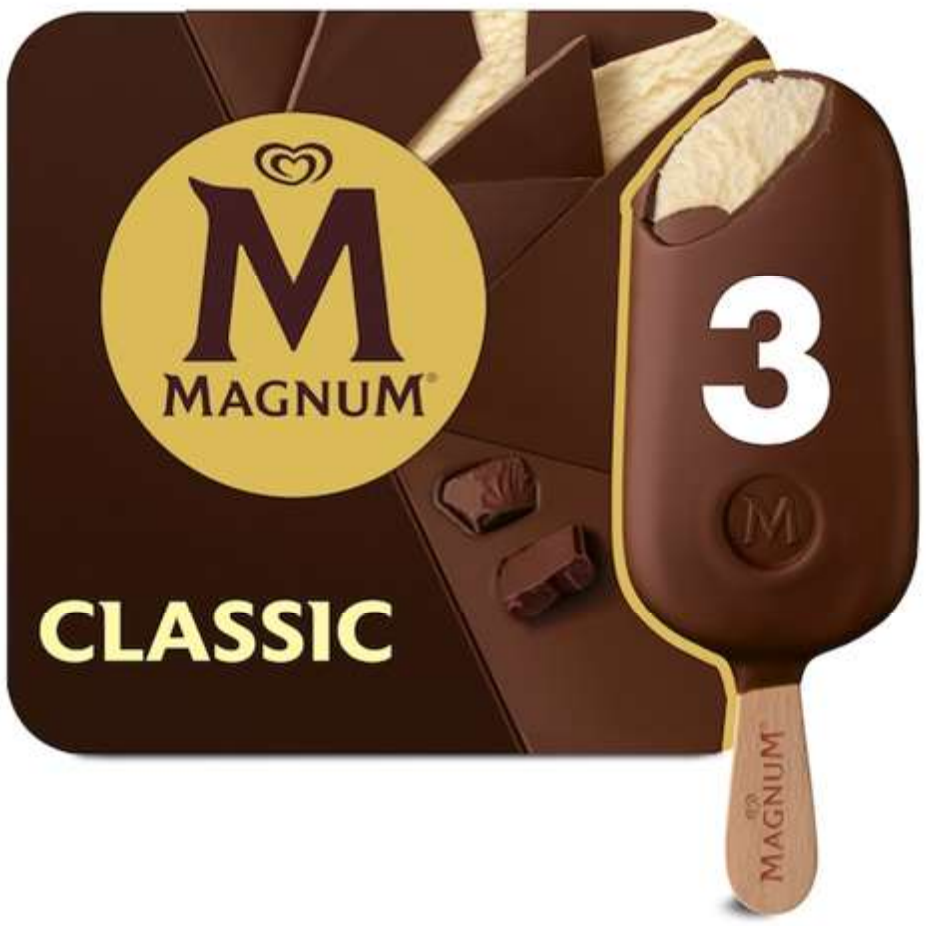

What is the maximum price that you would be willing to pay for 3 pieces vanilla ice cream coated with milk chocolate?

£0,00 £3,25 £6,50

Question 3 (11.3.2. Plant-based Ice cream) (Q107)

Plant-based ice cream

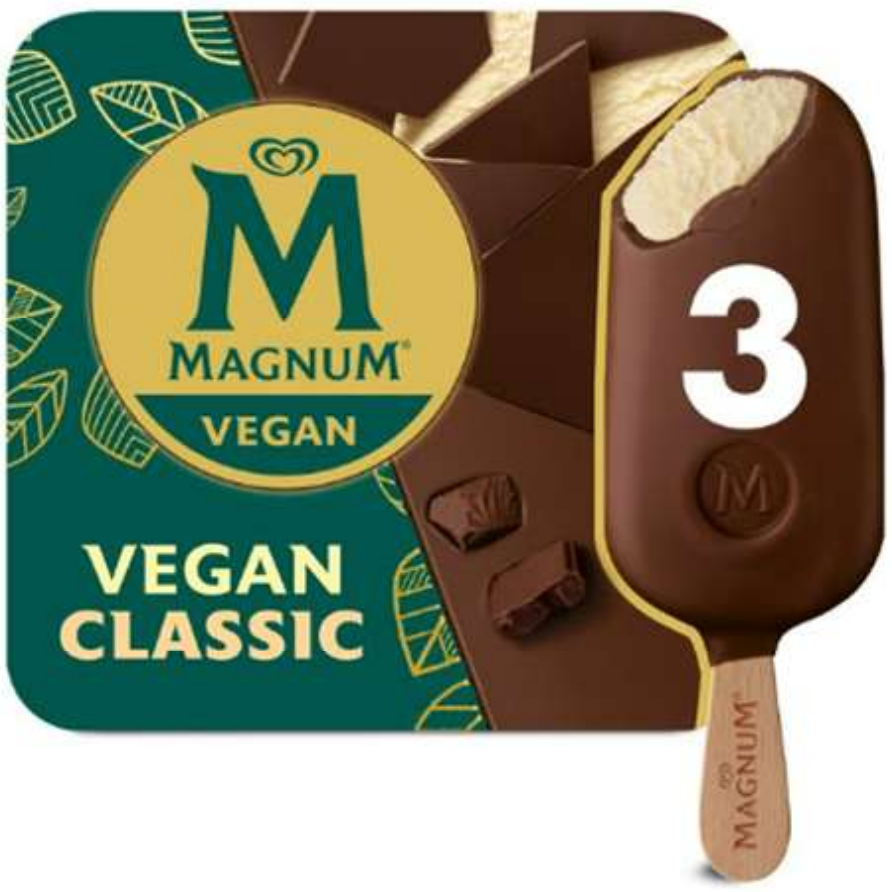

What is the maximum price that you would be willing to pay for 3 pieces plant-based vanilla ice cream coated with milk chocolate?

£0,00 £3.25 £6.50

Screen20 Randomgroup A

Only ask this screen when:

1 . 7.1.2 Consumption yoghurt Greater than Nooit of minder vaak dan eens per maand (1)

Question 1 (intro)

Imagine you are shopping in a grocery store and have decided to buy yoghurt.

Please indicate on the scales below (by sliding the marker to the left or to the right) for each of the products what is the maximum price that you usually would be willing to pay?

Question 2 (11.4.1. Dairy Yoghurt) (Q108)

Dairy-based yoghurt

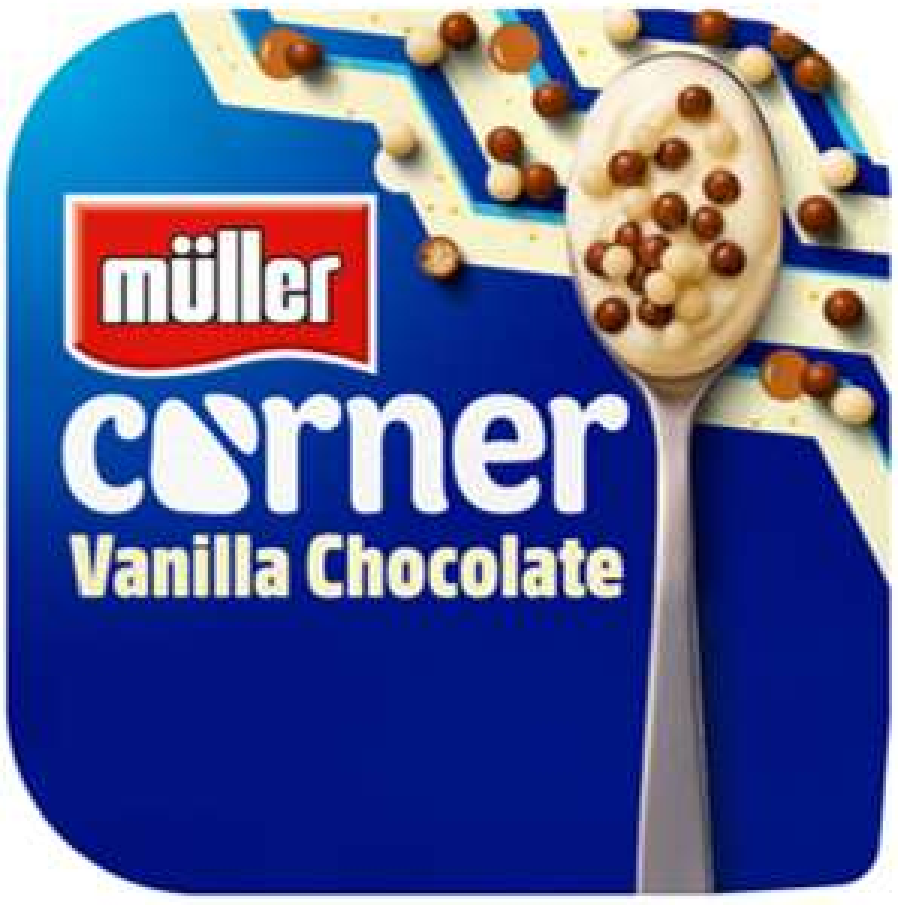

What is the maximum price that you would be willing to pay for 124 gram yoghurt?

£0,00 £0,90 £1,80

Question 3 (11.4.2. Plant-based Yoghurt) (Q109)

Plant-based yoghurt

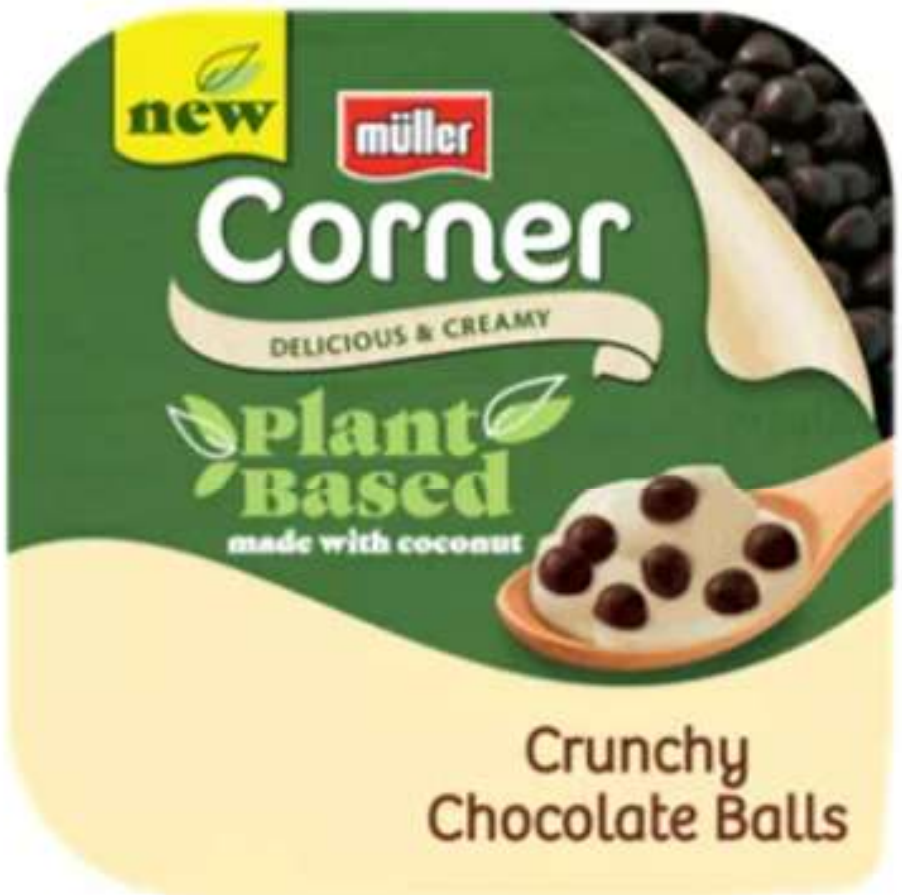

What is the maximum price that you would be willing to pay for 124 gram plant-based yoghurt?

£0,00 £0,90 £1.80

Screen21

Question 1 (intro)

You are almost at the end of the questionnaire. We have some last general questions:

Question 2 (Gender) (Q110)

What is your gender?

- ◯ Male (1)
- ◯ Female (2)
- ◯ I identify differently, namely (other)

Question 3 (Age) (Q111)

What is your age?

Question 4 (Educational level) (Q112)

Please select the highest level of education you have achieved to date:

- ◯ None (1)
- ◯ GCSE grades D and below or equivalent (2)
- ◯ GCSE grades A* - C or equivalent (3)
- ◯ GCE A-levels or equivalent (4)
- ◯ Undergraduate degree or equivalent (5)
- ◯ Postgraduate degree or equivalent (6)

Question 5 (Residence) (Q113)

Where do you live?

- ◯ In a big city (more than 100.000 inhabitants) (1)
- ◯ In a medium-sized city (50.000 - 100.000 inhabitants) (2)
- ◯ In a small city (20.000 - 50.000 inhabitants) (3)
- ◯ In a town (2.500 - 20.000 inhabitants) (4)
- ◯ In a small village (less than 2.500 inhabitants) (5)

Question 6 (Urban/rural) (Q114)

How would you describe the area where you live?

- ◯ Urban area (1)
- ◯ Rural area (2)

Question 7 (Region UK) (Q115)

In which region of the UK do you live?

- ◯ North-East (1)
- ◯ North-West (2)
- ◯ Yorkshire and the Humber (3)
- ◯ East Midlands (4)
- ◯ West Midlands (5)
- ◯ South West (6)
- ◯ South East (7)
- ◯ London (8)
- ◯ East of England (9)
- ◯ Northern Ireland (10)
- ◯ Wales (11)
- ◯ Scotland (12)

Screen22

ENDSCREEN

Question 1 (End)

This is the end of the questionnaire. You send the questionnaire by pressing the 'Send' button below.

Thank you for completing the questionnaire!
